# Supplementary material for: Air quality co-benefits for human health and agriculture counterbalance costs to meet Paris Agreement pledges
Source: Nat Commun. 2018 Nov 22;9:4939. doi: 10.1038/s41467-018-06885-9 (PMC6250710; doi:10.1038/s41467-018-06885-9)
Supplement: Supplementary file 1 — Supplementary Information [file 41467_2018_6885_MOESM1_ESM.pdf]

Air quality co-benefits for human health and agriculture counterbalance costs to meet Paris Agreement pledges

Vandyck et al.

Supplementary information

Supplementary figures

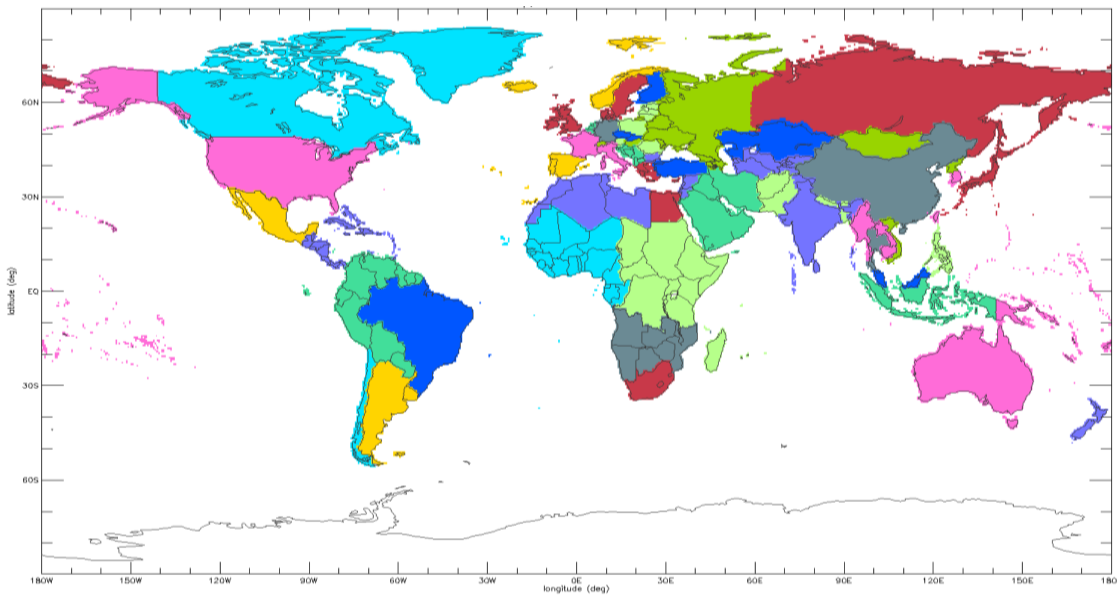

Supplementary Figure 1: The 56 continental emission source regions defined in TM5-FASST. Based on this regional aggregation, the model produces approximate resulting pollutant grid maps of $PM_{2.5}$ and ozone, using an implicit underlying spatial distribution of the emissions.

Supplementary tables

Supplementary Table 1: Total greenhouse gas emissions (including LULUCF) by region, scenario, and year. Source: POLES model.

Greenhouse gases (Mt CO_2e)	2010	Reference 2030	Reference 2050	NDC 2030	NDC 2050	2C 2030	2C 2050
World	44517	57359	65653	50761	42278	39562	17590
Algeria and Libya	253	458	641	428	532	355	182
Argentina	383	402	438	402	249	238	60
Australia	558	472	473	418	175	290	-48
Brazil	1279	1234	1315	1150	759	710	77
Canada	760	629	621	558	325	473	117
Chile	96	132	147	75	38	53	-17
China	10942	16676	16188	13817	8849	11474	4431
Iceland, Norway and Switzerland	77	79	54	72	52	72	26
Egypt	273	408	659	408	559	335	252
European Union (28)	4328	3212	2716	2885	2356	2882	984
India	2334	5070	7369	5074	5707	3633	1689
Indonesia	1920	1710	2123	1711	1713	1281	894
Iran	678	1124	1516	1122	1092	764	331
Japan	1067	902	783	907	491	776	220
Korea (Republic)	598	677	612	503	347	502	215
Malaysia	404	534	619	534	436	401	234
Mediterranean Middle-East	197	246	351	220	278	198	85
Mexico	718	752	796	623	571	594	289
New Zealand	42	57	65	24	20	23	2
Rest of Balkans	118	130	113	120	82	94	22
Rest of Central America & Carib.	322	388	444	385	265	174	100
Rest of CIS	739	1012	1287	948	1134	854	294
Rest of Pacific	102	127	178	127	138	84	56
Rest of Persian Gulf	685	1133	1376	1130	1019	824	385
Rest of South America	1122	1109	1197	1109	44	137	-593
Rest of South Asia	662	1225	1970	1226	1662	969	742
Rest of South-East Asia	949	1285	1393	1281	1000	791	387
Rest of Sub-Saharan Africa	1878	3522	5544	2708	3287	1625	1400
Russian Federation	1612	1948	2268	1948	1227	1116	402
Saudi Arabia	545	800	958	798	551	626	291
South Africa	511	641	771	523	543	460	200
Thailand	365	499	657	404	497	357	215
Tunisia, Morocco & West. Sahara	104	189	286	158	204	154	124
Turkey	309	531	738	532	406	320	98
Ukraine	311	301	351	300	243	179	68
United States	5691	5038	5171	3514	2348	3519	1358
Vietnam	273	514	764	514	601	346	214

Supplementary Table 2: Total primary energy demand in million tonnes of oil equivalent (Mtoe) by region, scenario, and year. Source: POLES model.

Total primary energy demand		Reference	Reference	NDC	NDC	2C	2C
Mtoe	2010	2030	2050	2030	2050	2030	2050
World	12530	16896	20535	16398	18394	15155	15054
Algeria and Libya	60	134	205	131	187	110	121
Argentina	80	95	121	95	119	89	98
Australia	124	138	168	138	153	134	132
Brazil	265	327	439	327	439	317	389
Canada	253	281	322	281	302	267	238
Chile	31	47	58	46	52	44	44
China	2628	4490	5076	4234	4215	3898	3756
Iceland, Norway and Switzerland	66	73	75	73	76	73	69
Egypt	73	119	207	119	198	107	149
European Union (28)	1736	1595	1541	1567	1490	1566	1216
India	689	1459	2228	1461	2045	1280	1528
Indonesia	202	327	488	327	447	295	310
Iran	208	394	558	394	440	282	295
Japan	500	431	409	436	370	412	347
Korea (Republic)	254	337	352	318	337	318	320
Malaysia	74	125	161	125	140	111	110
Mediterranean Middle-East	58	75	119	72	111	68	90
Mexico	176	241	331	236	327	232	292
New Zealand	20	24	30	26	29	27	27
Rest of Balkans	31	32	35	31	33	28	26
Rest of Central America & Carib.	80	122	176	122	170	115	143
Rest of CIS	188	306	425	295	403	277	236
Rest of Pacific	202	374	462	373	389	294	246
Rest of Persian Gulf	5	8	26	8	21	7	12
Rest of South America	154	219	344	220	332	201	266
Rest of South Asia	139	291	506	291	468	253	329
Rest of South-East Asia	223	324	393	324	378	300	313
Rest of Sub-Saharan Africa	323	546	896	544	876	525	752
Russian Federation	690	708	698	709	601	617	484
Saudi Arabia	186	263	330	263	237	212	192
South Africa	138	157	190	142	164	133	133
Thailand	113	170	214	163	201	156	155
Tunisia, Morocco & West. Sahara	27	51	80	47	70	46	58
Turkey	104	176	253	176	213	153	175
Ukraine	133	110	132	111	118	92	93
United States	2249	2279	2424	2136	2259	2132	2116
Vietnam	58	117	181	117	165	99	113

Supplementary Table 3: The share of fossil fuels in total primary energy demand by region, scenario, and year. Source: POLES model.

Fossil fuels*		Reference	Reference	NDC	NDC	2C	2C
Share in total primary energy (%)	2010	2030	2050	2030	2050	2030	2050
World	82	79	75	76	65	70	48
Algeria and Libya	100	99	98	99	96	98	78
Argentina	88	80	69	80	64	72	38
Australia	95	84	78	84	71	83	55
Brazil	54	50	48	50	45	45	28
Canada	73	59	49	57	37	52	23
Chile	78	71	65	68	55	62	41
China	88	83	76	78	56	71	41
Iceland, Norway and Switzerland	54	49	42	48	42	48	38
Egypt	96	94	91	94	89	90	74
European Union (28)	75	64	58	62	57	62	43
India	72	81	82	81	75	70	46
Indonesia	69	74	78	74	74	67	46
Iran	99	98	98	98	94	94	68
Japan	81	74	65	74	55	69	43
Korea (Republic)	81	75	66	63	48	63	45
Malaysia	96	94	90	94	83	87	61
Mediterranean Middle-East	97	93	82	91	77	88	53
Mexico	89	82	65	79	61	78	48
New Zealand	57	56	54	39	38	39	31
Rest of Balkans	85	78	67	77	61	71	33
Rest of Central America & Carib.	73	69	66	69	64	64	48
Rest of CIS	96	94	89	92	87	90	54
Rest of Pacific	99	99	98	99	96	97	79
Rest of Persian Gulf	52	67	84	67	79	58	45
Rest of South America	82	74	63	74	59	67	33
Rest of South Asia	59	82	86	82	83	76	60
Rest of South-East Asia	79	76	69	76	66	68	46
Rest of Sub-Saharan Africa	22	39	55	38	53	35	37
Russian Federation	91	85	80	85	66	72	39
Saudi Arabia	100	99	98	99	89	98	78
South Africa	90	87	83	82	71	78	43
Thailand	84	84	87	82	83	80	66
Tunisia, Morocco & West. Sahara	89	90	87	88	83	88	77
Turkey	90	76	74	76	62	68	45
Ukraine	81	74	72	74	63	59	35
United States	83	73	64	63	46	63	41
Vietnam	71	80	84	80	81	75	56

* Includes fossil fuels used in combination with carbon capture and storage.

Supplementary Table 4: The share of biomass in total primary energy demand by region, scenario, and year. Source: POLES model.

Biomass*		Reference	Reference	NDC	NDC	2C	2C
Share in total primary energy (%)	2010	2030	2050	2030	2050	2030	2050
World	10	10	10	11	13	12	20
Algeria and Libya	0	1	1	1	2	1	15
Argentina	7	8	11	8	13	11	27
Australia	3	8	11	9	15	10	31
Brazil	31	29	32	30	35	34	48
Canada	6	12	15	13	20	15	22
Chile	15	22	25	25	32	29	39
China	8	7	8	8	12	10	16
Iceland, Norway and Switzerland	6	5	7	6	7	6	11
Egypt	2	1	1	1	1	2	7
European Union (28)	8	14	17	15	17	15	21
India	25	13	8	13	9	16	19
Indonesia	26	18	13	18	15	21	35
Iran	0	1	1	1	2	1	17
Japan	2	5	9	5	13	8	22
Korea (Republic)	3	3	3	3	3	3	7
Malaysia	3	5	6	5	9	9	27
Mediterranean Middle-East	0	2	6	2	7	3	23
Mexico	5	7	12	7	12	8	20
New Zealand	6	5	6	6	8	6	14
Rest of Balkans	7	9	12	10	14	12	29
Rest of Central America & Carib.	21	16	17	17	18	18	30
Rest of CIS	1	4	6	4	6	5	26
Rest of Pacific	0	0	1	0	1	1	12
Rest of Persian Gulf	39	22	9	22	12	26	38
Rest of South America	9	15	22	15	24	19	42
Rest of South Asia	37	15	8	15	10	18	20
Rest of South-East Asia	10	10	10	10	11	12	21
Rest of Sub-Saharan Africa	75	57	37	58	38	60	51
Russian Federation	1	5	7	5	12	9	26
Saudi Arabia	0	0	1	0	7	0	17
South Africa	8	9	8	10	11	12	22
Thailand	15	12	9	14	10	16	23
Tunisia, Morocco & West. Sahara	8	5	3	5	3	5	4
Turkey	4	7	9	7	12	10	20
Ukraine	1	2	3	2	4	4	14
United States	5	10	16	13	21	12	23
Vietnam	24	13	10	13	10	17	23

* Includes biomass used in combination with carbon capture and storage.

Supplementary Table 5: The share of other low-carbon energy in total primary energy demand by region, scenario, and year. Source: POLES model.

Other low-carbon energy*<br>*Share in total primary energy (%)*	2010	Reference<br>2030	Reference<br>2050	NDC<br>2030	NDC<br>2050	2C<br>2030	2C<br>2050
World	9	12	15	14	22	17	33
Algeria and Libya	0	0	1	0	2	1	6
Argentina	6	10	19	10	23	16	35
Australia	2	7	11	7	14	8	15
Brazil	15	20	22	20	23	21	28
Canada	22	31	38	32	45	35	58
Chile	6	7	11	8	14	9	20
China	4	10	16	13	32	19	43
Iceland, Norway and Switzerland	39	48	53	49	53	49	53
Egypt	2	4	8	4	10	8	18
European Union (28)	17	23	24	23	26	23	35
India	3	6	10	6	16	14	35
Indonesia	5	8	9	8	11	12	19
Iran	0	1	1	1	4	5	15
Japan	17	21	26	20	32	23	35
Korea (Republic)	16	22	31	33	48	34	48
Malaysia	1	2	4	2	8	3	11
Mediterranean Middle-East	3	6	12	7	16	9	24
Mexico	6	12	23	14	27	15	32
New Zealand	37	39	40	55	54	55	55
Rest of Balkans	9	11	19	11	23	15	35
Rest of Central America & Carib.	6	14	17	15	18	17	22
Rest of CIS	3	3	5	4	7	5	20
Rest of Pacific	0	0	1	0	2	2	8
Rest of Persian Gulf	9	12	7	12	9	16	17
Rest of South America	12	14	17	14	20	17	28
Rest of South Asia	4	3	5	3	7	6	20
Rest of South-East Asia	10	14	21	14	24	20	34
Rest of Sub-Saharan Africa	3	5	8	6	9	6	12
Russian Federation	9	11	13	11	23	19	36
Saudi Arabia	0	1	1	1	4	1	5
South Africa	3	5	8	8	18	11	35
Thailand	0	3	3	4	6	4	9
Tunisia, Morocco & West. Sahara	2	5	9	5	12	6	17
Turkey	5	16	17	16	26	22	34
Ukraine	19	25	25	25	33	37	51
United States	12	17	20	24	33	24	36
Vietnam	4	6	6	6	8	9	20

* Other low-carbon energy includes nuclear, hydro and other renewables.

Supplementary Table 6: The share of GHG emission reductions through LULUCF measures in total mitigation. Source: POLES and GLOBIOM.

LULUCF mitigation<br>*Share in total GHG mitigation (%)*	NDC<br>2010-2030	NDC<br>2030-2050	2C<br>2010-2030	2C<br>2030-2050
World	18	19	23	14
Algeria and Libya	1	1	1	1
Argentina	0	61	53	38
Australia	93	71	84	52
Brazil	50	57	44	35
Canada	70	29	42	16
Chile	70	53	58	41
China	3	-1	2	0
Iceland, Norway and Switzerland	51	53	47	-8
Egypt	0	0	0	0
European Union (28)	24	22	24	8
India	0	3	5	2
Indonesia	0	45	41	23
Iran	0	2	2	1
Japan	0	0	0	-6
Korea (Republic)	0	-1	0	-1
Malaysia	0	31	32	17
Mediterranean Middle-East	1	1	1	0
Mexico	66	47	57	14
New Zealand	26	25	26	22
Rest of Balkans	31	19	21	8
Rest of Central America & Carib.	0	69	70	41
Rest of CIS	3	2	1	0
Rest of Pacific	0	0	0	0
Rest of Persian Gulf	0	50	81	57
Rest of South America	0	84	80	66
Rest of South Asia	0	-1	6	1
Rest of South-East Asia	0	39	33	24
Rest of Sub-Saharan Africa	80	78	76	64
Russian Federation	0	11	8	6
Saudi Arabia	0	1	1	0
South Africa	10	3	10	2
Thailand	28	20	21	9
Tunisia, Morocco & West. Sahara	10	5	11	4
Turkey	0	18	29	12
Ukraine	0	3	2	1
United States	7	7	7	5
Vietnam	0	18	17	7

Supplementary Table 7: SO_2 emissions by region, climate scenario, and year under Fixed air pollution legislation. Source: POLES model.

SO_2 (kt)		Reference	Reference	INDC	INDC	2C	2C
Fixed air pollution legislation	2010	2030	2050	2030	2050	2030	2050
World	94762	102607	116814	95593	81273	77808	48856
Algeria and Libya	411	464	497	441	442	385	278
Argentina	556	505	678	500	543	439	393
Australia	1492	1281	1140	1266	931	1171	907
Brazil	1175	1269	1623	1250	1491	1102	963
Canada	1994	4341	8220	3804	3348	2554	1919
Chile	402	526	597	480	420	416	281
China	28930	26636	23663	23417	13866	19490	9112
Iceland, Norway and Switzerland	142	80	52	77	50	75	40
Egypt	531	524	918	521	740	458	422
European Union (28)	6236	3187	2449	3016	2231	2923	1715
India	7852	15746	19594	15732	13795	11062	4015
Indonesia	2104	1902	2063	1899	1906	1762	1417
Iran	1152	1909	3012	1899	1945	1291	1040
Japan	1529	1089	967	1078	844	988	880
Korea (Republic)	957	883	759	718	560	696	508
Malaysia	556	720	809	715	555	563	390
Mediterranean Middle-East	748	914	995	834	791	736	432
Mexico	937	943	923	897	803	860	591
New Zealand	143	124	106	108	86	107	99
Rest of Balkans	398	248	197	236	132	157	35
Rest of Central America & Carib.	898	864	1107	846	925	687	447
Rest of CIS	2621	3679	4058	3330	3228	2936	1655
Rest of Pacific	65	65	164	65	134	57	97
Rest of Persian Gulf	1812	2312	2810	2292	2381	1996	1624
Rest of South America	1403	1721	2525	1695	2121	1366	1396
Rest of South Asia	1178	2107	3050	2096	2537	1910	1350
Rest of South-East Asia	2229	2314	2232	2274	1875	1818	1157
Rest of Sub-Saharan Africa	4281	5901	8724	5770	7651	5202	5212
Russian Federation	3948	4918	4652	4906	3277	3793	3921
Saudi Arabia	2066	2103	1775	2085	1331	1766	842
South Africa	3127	2578	2672	2182	1777	1881	776
Thailand	643	741	902	650	701	584	476
Tunisia, Morocco & West. Sahara	313	504	717	392	438	369	217
Turkey	1419	1866	2778	1862	1452	1009	476
Ukraine	1546	1146	1296	1144	649	425	164
United States	8477	5611	6764	4226	4255	4116	3178
Vietnam	489	889	1326	889	1062	659	430

Supplementary Table 8: SO_2 emissions by region, climate scenario, and year under Stringent air pollution legislation. Source: POLES model.

SO_2 (kt)		Reference	Reference	INDC	INDC	2C	2C
Stringent air pollution legislation	2010	2030	2050	2030	2050	2030	2050
World	94762	79152	51369	74465	37729	60517	26142
Algeria and Libya	411	319	249	302	214	256	125
Argentina	556	414	300	411	250	364	205
Australia	1492	1110	510	1106	440	1054	446
Brazil	1175	1023	740	1009	684	886	475
Canada	1994	4201	4808	3679	2143	2461	1392
Chile	402	439	258	403	192	353	141
China	28930	15846	10097	14093	6278	11903	3957
Iceland, Norway and Switzerland	142	50	31	48	30	47	27
Egypt	531	356	354	355	307	308	174
European Union (28)	6236	1953	1200	1861	1087	1828	897
India	7852	14489	5420	14488	4035	10057	1369
Indonesia	2104	1554	965	1554	914	1469	761
Iran	1152	1441	1192	1437	824	972	481
Japan	1529	758	587	754	538	709	655
Korea (Republic)	957	646	345	510	260	504	276
Malaysia	556	553	281	552	187	433	123
Mediterranean Middle-East	748	743	348	675	291	594	188
Mexico	937	659	285	629	257	606	210
New Zealand	143	112	47	97	39	96	46
Rest of Balkans	398	92	49	89	39	68	20
Rest of Central America & Carib.	898	635	285	624	263	500	159
Rest of CIS	2621	3585	1474	3247	1210	2863	735
Rest of Pacific	65	56	68	57	57	50	43
Rest of Persian Gulf	1812	1723	1321	1714	1127	1488	726
Rest of South America	1403	1320	1110	1312	959	1084	714
Rest of South Asia	1178	1747	508	1739	438	1569	258
Rest of South-East Asia	2229	1788	908	1776	776	1416	500
Rest of Sub-Saharan Africa	4281	5440	7792	5320	6738	4788	4576
Russian Federation	3948	4616	2577	4611	2096	3543	3049
Saudi Arabia	2066	1425	600	1420	453	1211	315
South Africa	3127	1624	893	1453	768	1322	600
Thailand	643	604	343	531	275	480	195
Tunisia, Morocco & West. Sahara	313	401	235	294	174	277	99
Turkey	1419	1686	1148	1684	624	885	229
Ukraine	1546	634	307	633	182	298	56
United States	8477	4390	3254	3275	2198	3238	1765
Vietnam	489	721	478	723	384	538	155

Supplementary Table 9: SO_2 emissions by region, climate scenario, and year under Best available air pollution abatement technologies. Source: POLES model.

SO_2 (kt) *Best available abatement technologies*	2010	Reference 2030	Reference 2050	INDC 2030	INDC 2050	2C 2030	2C 2050
World	94762	27662	29354	25941	22311	22610	17429
Algeria and Libya	411	76	77	73	66	58	39
Argentina	556	175	226	174	193	160	182
Australia	1492	373	319	369	283	346	301
Brazil	1175	403	477	399	450	367	365
Canada	1994	2117	3703	1910	1772	1423	1223
Chile	402	179	196	167	153	151	122
China	28930	7194	6019	6323	3593	5237	2241
Iceland, Norway and Switzerland	142	35	27	34	27	33	24
Egypt	531	112	147	111	132	96	81
European Union (28)	6236	1184	979	1136	898	1109	746
India	7852	1476	1552	1472	1248	1113	615
Indonesia	2104	416	413	416	399	400	353
Iran	1152	422	726	419	537	313	348
Japan	1529	406	354	403	302	366	270
Korea (Republic)	957	268	221	223	170	217	147
Malaysia	556	109	112	108	93	91	72
Mediterranean Middle-East	748	190	208	175	181	156	136
Mexico	937	172	173	166	161	161	149
New Zealand	143	37	30	33	26	32	31
Rest of Balkans	398	35	27	34	23	26	12
Rest of Central America & Carib.	898	107	129	105	119	93	86
Rest of CIS	2621	566	534	520	462	471	318
Rest of Pacific	65	18	40	18	34	16	28
Rest of Persian Gulf	1812	672	931	665	808	593	526
Rest of South America	1403	567	845	560	746	484	636
Rest of South Asia	1178	167	234	165	210	148	136
Rest of South-East Asia	2229	520	445	509	409	443	322
Rest of Sub-Saharan Africa	4281	3885	4598	3860	4382	3724	3793
Russian Federation	3948	2039	1619	2035	1415	1807	1794
Saudi Arabia	2066	370	367	365	294	305	221
South Africa	3127	835	720	780	640	732	562
Thailand	643	157	151	144	132	132	101
Tunisia, Morocco & West. Sahara	313	61	74	53	58	49	38
Turkey	1419	414	507	413	311	257	152
Ukraine	1546	86	93	86	59	45	22
United States	8477	1654	1890	1355	1365	1323	1140
Vietnam	489	166	190	165	164	132	97

Supplementary Table 10: NO_x emissions by region, climate scenario, and year under Fixed air pollution legislation. Source: POLES model.

NO_x (kt)		Reference	Reference	INDC	INDC	2C	2C
Fixed air pollution legislation	2010	2030	2050	2030	2050	2030	2050
World	132434	162031	182948	155651	157906	140359	116090
Algeria and Libya	1220	2266	2825	2197	2624	1829	1547
Argentina	1104	1158	1242	1155	1146	1036	797
Australia	2024	1829	1814	1792	1429	1576	1081
Brazil	3886	4113	4525	4091	4363	3808	3357
Canada	1780	2632	3813	2425	2161	1859	1245
Chile	483	596	600	573	520	534	394
China	27828	40600	41662	37636	32134	33582	23657
Iceland, Norway and Switzerland	415	286	193	277	190	271	163
Egypt	1139	1743	2765	1740	2616	1519	1748
European Union (28)	12075	9316	8699	8993	7940	8747	5519
India	7916	16351	21589	16313	19464	14050	12590
Indonesia	3147	3753	4613	3749	4383	3446	3140
Iran	2048	2755	3362	2740	2883	2160	1799
Japan	3181	2413	1952	2409	1675	2175	1447
Korea (Republic)	2219	2357	1995	1956	1500	1896	1319
Malaysia	886	1391	1737	1382	1548	1227	1244
Mediterranean Middle-East	653	930	1154	873	1033	788	717
Mexico	2179	2237	2577	2151	2382	2082	1906
New Zealand	179	188	204	159	155	155	134
Rest of Balkans	256	184	176	181	160	158	116
Rest of Central America & Carib.	1084	1349	1700	1338	1630	1219	1227
Rest of CIS	1429	1942	2312	1866	2152	1737	1281
Rest of Pacific	59	84	156	84	146	77	112
Rest of Persian Gulf	2490	4025	4523	3981	4028	3286	2663
Rest of South America	2736	3523	3921	3463	3654	3005	2694
Rest of South Asia	1604	4133	6935	4129	6699	3751	4761
Rest of South-East Asia	2981	3878	3650	3770	3468	3373	2815
Rest of Sub-Saharan Africa	15184	15805	19310	15728	19047	15357	16532
Russian Federation	5027	4571	4467	4554	3290	3630	1977
Saudi Arabia	2157	2954	3242	2905	2565	2306	1784
South Africa	1706	1953	2226	1792	1790	1585	1200
Thailand	1417	1856	2177	1752	1926	1624	1472
Tunisia, Morocco & West. Sahara	702	1233	1723	1113	1411	1073	1072
Turkey	1280	1879	2285	1872	1899	1609	1370
Ukraine	862	661	751	660	616	493	361
United States	16334	13719	14061	12493	11427	12170	9533
Vietnam	763	1366	2011	1360	1855	1169	1315

Supplementary Table 11: NO_x emissions by region, climate scenario, and year under Stringent air pollution legislation. Source: POLES model.

NO_x (kt)		Reference	Reference	INDC	INDC	2C	2C
Stringent air pollution legislation	2010	2030	2050	2030	2050	2030	2050
World	132434	122309	104923	118244	91955	106708	70747
Algeria and Libya	1220	1867	1424	1804	1302	1490	808
Argentina	1104	997	767	995	709	887	518
Australia	2024	1452	1090	1421	827	1245	626
Brazil	3886	3178	2569	3161	2477	2949	2039
Canada	1780	2158	3125	1958	1545	1431	807
Chile	483	484	340	465	300	432	233
China	27828	21294	14992	20065	11534	18224	8868
Iceland, Norway and Switzerland	415	207	124	200	121	196	112
Egypt	1139	1463	1566	1460	1470	1274	999
European Union (28)	12075	6667	5358	6459	4965	6265	3732
India	7916	15194	13135	15164	11921	13100	8160
Indonesia	3147	3193	2901	3189	2744	2930	2064
Iran	2048	2378	1827	2365	1601	1843	1079
Japan	3181	1778	1304	1761	1123	1589	1000
Korea (Republic)	2219	1847	1085	1497	852	1448	760
Malaysia	886	1199	1227	1191	1109	1054	907
Mediterranean Middle-East	653	797	623	746	558	670	420
Mexico	2179	1884	1457	1809	1345	1751	1111
New Zealand	179	139	109	115	81	112	73
Rest of Balkans	256	129	89	126	82	110	71
Rest of Central America & Carib.	1084	1147	1041	1138	1006	1035	789
Rest of CIS	1429	1807	1239	1736	1153	1614	767
Rest of Pacific	59	69	100	69	93	63	77
Rest of Persian Gulf	2490	3459	2529	3423	2286	2808	1596
Rest of South America	2736	2846	2306	2797	2141	2421	1685
Rest of South Asia	1604	3758	4042	3755	3868	3415	2746
Rest of South-East Asia	2981	3398	2706	3310	2609	2963	2208
Rest of Sub-Saharan Africa	15184	14868	17149	14802	16900	14487	14902
Russian Federation	5027	3727	2313	3712	1682	2933	1080
Saudi Arabia	2157	2463	1870	2423	1550	1931	1100
South Africa	1706	1614	1237	1467	970	1288	685
Thailand	1417	1652	1584	1557	1413	1442	1104
Tunisia, Morocco & West. Sahara	702	1126	1129	1011	902	975	681
Turkey	1280	1423	1219	1417	995	1185	729
Ukraine	862	511	488	511	404	401	252
United States	16334	8921	7431	7956	5979	7709	4974
Vietnam	763	1214	1429	1210	1340	1038	984

Supplementary Table 12: NO_x emissions by region, climate scenario, and year under Best available air pollution abatement technologies. Source: POLES model.

NO_x (kt)		Reference	Reference	INDC	INDC	2C	2C
Best available abatement technologies	2010	2030	2050	2030	2050	2030	2050
World	132434	63165	64742	60616	56013	55244	46068
Algeria and Libya	1220	304	334	292	302	245	240
Argentina	1104	373	403	370	384	348	332
Australia	2024	929	865	897	638	749	477
Brazil	3886	1283	1310	1273	1298	1235	1283
Canada	1780	1651	2836	1448	1280	963	595
Chile	483	182	182	175	162	163	130
China	27828	14283	13369	13318	10536	11897	8217
Iceland, Norway and Switzerland	415	141	82	137	79	132	73
Egypt	1139	327	537	321	505	288	389
European Union (28)	12075	4626	3913	4477	3678	4288	2791
India	7916	4226	5183	4184	4620	3539	3231
Indonesia	3147	1396	1495	1389	1440	1320	1222
Iran	2048	683	852	667	747	552	651
Japan	3181	1441	1143	1421	1000	1268	903
Korea (Republic)	2219	1038	826	898	676	850	596
Malaysia	886	490	584	482	533	432	455
Mediterranean Middle-East	653	277	359	257	315	228	263
Mexico	2179	580	718	563	679	540	618
New Zealand	179	69	71	61	58	58	53
Rest of Balkans	256	64	51	63	47	55	39
Rest of Central America & Carib.	1084	432	541	424	520	386	427
Rest of CIS	1429	403	478	387	441	362	348
Rest of Pacific	59	47	81	47	74	43	59
Rest of Persian Gulf	2490	1509	1594	1472	1444	1258	1100
Rest of South America	2736	1297	1363	1243	1249	1047	1099
Rest of South Asia	1604	766	1209	757	1129	673	822
Rest of South-East Asia	2981	2152	1752	2063	1696	1885	1518
Rest of Sub-Saharan Africa	15184	11887	11105	11843	11026	11765	10675
Russian Federation	5027	1224	1324	1205	931	1003	686
Saudi Arabia	2157	982	989	940	941	762	701
South Africa	1706	675	753	614	558	504	421
Thailand	1417	606	614	582	575	547	493
Tunisia, Morocco & West. Sahara	702	242	335	218	288	203	214
Turkey	1280	824	894	817	737	692	569
Ukraine	862	184	193	183	161	148	125
United States	16334	5017	5666	4578	4585	4337	3744
Vietnam	763	554	736	548	682	476	510

Supplementary Table 13: NH_3 emissions by region, climate scenario, and year under Fixed air pollution legislation. Source: POLES model.

NH_3 (kt)		Reference	Reference	INDC	INDC	2C	2C
Fixed air pollution legislation	2010	2030	2050	2030	2050	2030	2050
World	60536	73008	82497	68495	70171	60793	50119
Algeria and Libya	361	645	799	627	752	562	533
Argentina	1701	1965	2202	1965	1735	1557	1396
Australia	792	764	776	764	680	733	597
Brazil	2880	3480	3792	3385	3182	2619	2279
Canada	631	668	764	663	688	651	676
Chile	389	501	559	430	414	402	362
China	14087	18254	18976	15452	14884	14363	8385
Iceland, Norway and Switzerland	119	145	158	138	158	134	120
Egypt	437	717	921	717	861	634	617
European Union (28)	3651	3572	3732	3110	3282	3108	2773
India	7529	10020	12806	10021	11364	8070	6451
Indonesia	1703	2195	2625	2195	2197	1762	1524
Iran	182	284	349	284	329	247	234
Japan	268	278	274	275	213	228	207
Korea (Republic)	198	233	254	216	231	216	191
Malaysia	310	451	567	451	428	345	298
Mediterranean Middle-East	38	24	31	24	30	23	26
Mexico	840	977	1100	908	989	879	777
New Zealand	148	162	169	133	126	133	111
Rest of Balkans	68	86	96	85	89	77	77
Rest of Central America & Carib.	476	656	821	656	646	488	403
Rest of CIS	750	855	901	843	885	815	728
Rest of Pacific	31	37	40	37	36	32	31
Rest of Persian Gulf	169	115	147	115	141	103	106
Rest of South America	347	367	384	367	344	328	310
Rest of South Asia	2729	3494	4670	3494	4487	3131	3311
Rest of South-East Asia	1491	2068	2558	2068	2133	1632	1451
Rest of Sub-Saharan Africa	11015	11147	11991	10669	9972	9587	8734
Russian Federation	757	919	1018	919	954	871	804
Saudi Arabia	620	1029	1268	1028	1097	884	812
South Africa	373	424	447	400	418	378	350
Thailand	820	1120	1381	938	1108	845	700
Tunisia, Morocco & West. Sahara	26	38	47	38	47	37	47
Turkey	497	734	957	734	756	597	499
Ukraine	270	328	347	328	332	305	266
United States	3749	4150	4436	3913	4050	3909	3800
Vietnam	87	108	131	108	132	107	135

Supplementary Table 14: NH_3 emissions by region, climate scenario, and year under Stringent air pollution legislation. Source: POLES model.

NH_3 (kt)		Reference	Reference	INDC	INDC	2C	2C
Stringent air pollution legislation	2010	2030	2050	2030	2050	2030	2050
World	60536	73008	64055	68495	55143	60793	40448
Algeria and Libya	361	645	705	627	664	562	472
Argentina	1701	1965	1734	1965	1370	1557	1104
Australia	792	764	590	764	519	733	458
Brazil	2880	3480	2882	3385	2431	2619	1762
Canada	631	668	485	663	437	651	429
Chile	389	501	440	430	326	402	285
China	14087	18254	10269	15452	8206	14363	4921
Iceland, Norway and Switzerland	119	145	124	138	123	134	94
Egypt	437	717	815	717	763	634	550
European Union (28)	3651	3572	2902	3110	2560	3108	2152
India	7529	10020	11420	10021	10158	8070	5862
Indonesia	1703	2195	2282	2195	1926	1762	1365
Iran	182	284	211	284	200	247	146
Japan	268	278	178	275	146	228	144
Korea (Republic)	198	233	170	216	156	216	132
Malaysia	310	451	507	451	385	345	271
Mediterranean Middle-East	38	24	23	24	23	23	22
Mexico	840	977	793	908	718	879	574
New Zealand	148	162	125	133	93	133	82
Rest of Balkans	68	86	71	85	67	77	59
Rest of Central America & Carib.	476	656	739	656	584	488	369
Rest of CIS	750	855	772	843	759	815	625
Rest of Pacific	31	37	30	37	28	32	24
Rest of Persian Gulf	169	115	96	115	94	103	74
Rest of South America	347	367	333	367	302	328	275
Rest of South Asia	2729	3494	4208	3494	4047	3131	3009
Rest of South-East Asia	1491	2068	2313	2068	1937	1632	1338
Rest of Sub-Saharan Africa	11015	11147	11495	10669	9605	9587	8447
Russian Federation	757	919	808	919	757	871	632
Saudi Arabia	620	1029	746	1028	648	884	481
South Africa	373	424	344	400	322	378	271
Thailand	820	1120	1231	938	990	845	630
Tunisia, Morocco & West. Sahara	26	38	46	38	46	37	46
Turkey	497	734	723	734	573	597	380
Ukraine	270	328	317	328	304	305	245
United States	3749	4150	2998	3913	2746	3909	2584
Vietnam	87	108	130	108	131	107	134

Supplementary Table 15: NH_3 emissions by region, climate scenario, and year under Best available air pollution abatement technologies. Source: POLES model.

NH_3 (kt)<br>*Best available abatement technologies*	2010	Reference 2030	Reference 2050	INDC 2030	INDC 2050	2C 2030	2C 2050
World	60536	42648	47944	40594	41693	36511	32120
Algeria and Libya	361	319	397	311	376	281	277
Argentina	1701	1374	1537	1374	1215	1092	981
Australia	792	519	522	519	461	499	407
Brazil	2880	2340	2538	2279	2147	1787	1567
Canada	631	341	387	338	350	332	343
Chile	389	348	389	299	288	280	252
China	14087	7171	7503	6214	6085	5828	3820
Iceland, Norway and Switzerland	119	101	109	96	109	94	84
Egypt	437	362	473	362	446	323	336
European Union (28)	3651	2466	2556	2156	2252	2155	1867
India	7529	5472	7018	5472	6325	4535	3984
Indonesia	1703	1103	1235	1103	1097	964	880
Iran	182	130	159	130	152	114	114
Japan	268	152	147	151	124	129	124
Korea (Republic)	198	135	145	127	134	127	114
Malaysia	310	265	329	265	256	209	187
Mediterranean Middle-East	38	15	19	15	19	15	19
Mexico	840	587	660	549	600	533	486
New Zealand	148	105	109	86	82	86	72
Rest of Balkans	68	57	62	56	58	51	52
Rest of Central America & Carib.	476	387	482	387	388	297	259
Rest of CIS	750	670	706	661	695	639	573
Rest of Pacific	31	25	27	25	25	21	22
Rest of Persian Gulf	169	56	74	56	73	51	60
Rest of South America	347	296	305	296	278	270	256
Rest of South Asia	2729	2021	2746	2021	2653	1836	2054
Rest of South-East Asia	1491	1303	1581	1303	1356	1072	1000
Rest of Sub-Saharan Africa	11015	9300	9830	8956	8380	8179	7496
Russian Federation	757	667	739	667	693	632	576
Saudi Arabia	620	459	565	458	494	395	368
South Africa	373	294	307	278	287	263	243
Thailand	820	645	782	549	638	500	422
Tunisia, Morocco & West. Sahara	26	33	41	33	42	33	43
Turkey	497	511	664	511	527	417	351
Ukraine	270	216	224	216	216	202	176
United States	3749	2298	2452	2169	2250	2167	2126
Vietnam	87	104	126	104	126	104	130

Supplementary Table 16: Non-Methane Volatile Organic Compounds emissions by region, climate scenario, and year under Fixed air pollution legislation. Source: POLES model.

NMVOC (kt)		Reference	Reference	INDC	INDC	2C	2C
Fixed air pollution legislation	2010	2030	2050	2030	2050	2030	2050
World	140372	178080	218894	177599	213825	172801	195408
Algeria and Libya	1069	2036	2476	1968	2269	1640	1339
Argentina	1180	1148	1208	1148	1195	1132	1114
Australia	951	949	971	947	941	934	926
Brazil	3921	3745	4087	3736	4047	3627	3683
Canada	1076	1101	1165	1105	1109	1082	1041
Chile	566	612	624	608	607	596	555
China	23580	39146	42957	38879	41733	38359	38856
Iceland, Norway and Switzerland	509	522	508	516	511	516	472
Egypt	1013	1575	2618	1576	2578	1481	2192
European Union (28)	7700	7815	8419	7746	8152	7721	7371
India	11449	17690	22845	17692	22463	17232	20688
Indonesia	6572	8042	9479	8050	9420	7820	8452
Iran	2023	3195	4370	3198	3528	2447	2474
Japan	1528	1618	1785	1629	1735	1621	1713
Korea (Republic)	995	1287	1258	1248	1201	1245	1176
Malaysia	1017	1594	1943	1595	1919	1550	1744
Mediterranean Middle-East	700	659	759	646	741	621	615
Mexico	2097	2419	3061	2388	3015	2360	2763
New Zealand	96	108	120	101	108	101	105
Rest of Balkans	216	271	350	275	363	284	295
Rest of Central America & Carib.	1384	1510	1807	1509	1803	1462	1629
Rest of CIS	1698	2499	3218	2489	3210	2452	2986
Rest of Pacific	121	118	152	118	150	113	133
Rest of Persian Gulf	1870	2688	3007	2689	2814	2411	2243
Rest of South America	3644	4053	4840	4055	4748	3794	3954
Rest of South Asia	3205	4952	8862	4956	8862	4859	8025
Rest of South-East Asia	4435	4933	5337	4935	5320	4798	4902
Rest of Sub-Saharan Africa	32971	38153	54090	38145	54020	37708	51356
Russian Federation	4518	4031	3582	4038	3360	3771	2689
Saudi Arabia	2074	2047	2222	2048	1791	1731	1437
South Africa	1396	1575	1665	1558	1643	1537	1450
Thailand	1826	2246	2526	2216	2487	2176	2228
Tunisia, Morocco & West. Sahara	591	848	1118	790	988	772	808
Turkey	730	921	1055	922	981	866	823
Ukraine	798	653	774	653	764	630	677
United States	9513	9248	10664	9352	10287	9307	9769
Vietnam	1341	2072	2973	2073	2963	2046	2728

Supplementary Table 17: Non-Methane Volatile Organic Compounds emissions by region, climate scenario, and year under Stringent air pollution legislation. Source: POLES model.

NMVOC (kt)		Reference	Reference	INDC	INDC	2C	2C
Stringent air pollution legislation	2010	2030	2050	2030	2050	2030	2050
World	140372	160228	152718	159787	149509	155845	139594
Algeria and Libya	1069	1534	1325	1474	1181	1216	731
Argentina	1180	967	764	967	755	959	717
Australia	951	835	651	833	633	822	626
Brazil	3921	3404	2718	3396	2689	3312	2497
Canada	1076	960	677	960	650	944	609
Chile	566	479	317	477	310	469	288
China	23580	35464	24871	35264	24263	34877	22963
Iceland, Norway and Switzerland	509	407	304	402	307	402	283
Egypt	1013	1312	1714	1313	1693	1249	1551
European Union (28)	7700	5836	4562	5793	4454	5775	4062
India	11449	16329	17603	16330	17301	15946	16407
Indonesia	6572	6856	5927	6862	5875	6705	5532
Iran	2023	2897	3255	2899	2536	2195	1762
Japan	1528	1515	1251	1520	1221	1515	1217
Korea (Republic)	995	1202	805	1168	773	1165	763
Malaysia	1017	1399	744	1400	738	1364	724
Mediterranean Middle-East	700	553	474	543	465	522	392
Mexico	2097	2150	1855	2124	1824	2101	1700
New Zealand	96	92	70	86	63	86	62
Rest of Balkans	216	195	180	198	190	206	146
Rest of Central America & Carib.	1384	1234	1205	1233	1200	1200	1120
Rest of CIS	1698	2265	1947	2257	1941	2226	1775
Rest of Pacific	121	92	75	92	74	88	66
Rest of Persian Gulf	1870	2402	2274	2402	2101	2164	1675
Rest of South America	3644	3325	2886	3325	2807	3115	2337
Rest of South Asia	3205	4596	5908	4598	5903	4522	5639
Rest of South-East Asia	4435	4649	3650	4650	3634	4525	3442
Rest of Sub-Saharan Africa	32971	37048	49026	37044	48959	36662	46804
Russian Federation	4518	3560	2211	3565	2068	3334	1683
Saudi Arabia	2074	1723	1483	1724	1209	1482	986
South Africa	1396	1205	812	1194	793	1180	735
Thailand	1826	2023	1179	1998	1147	1963	1089
Tunisia, Morocco & West. Sahara	591	789	827	735	725	717	594
Turkey	730	804	627	804	584	752	501
Ukraine	798	556	602	556	593	537	535
United States	9513	7653	6247	7685	6166	7652	5986
Vietnam	1341	1916	1693	1916	1682	1895	1596

Supplementary Table 18: Non-Methane Volatile Organic Compounds emissions by region, climate scenario, and year under Best available air pollution abatement technologies. Source: POLES model.

NMVOC (kt)		Reference	Reference	INDC	INDC	2C	2C
Best available abatement technologies	2010	2030	2050	2030	2050	2030	2050
World	140372	80700	91355	80470	89168	78331	82563
Algeria and Libya	1069	761	893	737	819	628	505
Argentina	1180	564	539	564	535	560	515
Australia	951	579	543	578	526	569	510
Brazil	3921	1933	1900	1926	1879	1870	1697
Canada	1076	476	462	476	441	463	405
Chile	566	192	190	192	186	188	172
China	23580	14973	16309	14877	15907	14680	15047
Iceland, Norway and Switzerland	509	336	329	332	332	332	306
Egypt	1013	459	756	459	745	437	653
European Union (28)	7700	4248	4570	4216	4490	4203	4108
India	11449	6579	8590	6578	8517	6477	8254
Indonesia	6572	3027	3174	3028	3155	2990	2991
Iran	2023	1894	2574	1895	1948	1409	1298
Japan	1528	828	868	832	848	824	834
Korea (Republic)	995	571	554	553	534	551	529
Malaysia	1017	345	394	345	391	342	378
Mediterranean Middle-East	700	359	357	353	352	340	292
Mexico	2097	1029	1183	1013	1164	999	1056
New Zealand	96	50	52	46	48	46	47
Rest of Balkans	216	134	187	138	200	150	148
Rest of Central America & Carib.	1384	441	506	440	505	427	470
Rest of CIS	1698	940	1123	937	1118	924	997
Rest of Pacific	121	36	54	37	53	34	47
Rest of Persian Gulf	1870	1786	1923	1786	1761	1590	1371
Rest of South America	3644	1742	1947	1741	1882	1600	1511
Rest of South Asia	3205	1183	2194	1183	2189	1172	2113
Rest of South-East Asia	4435	2269	2249	2266	2242	2228	2152
Rest of Sub-Saharan Africa	32971	22903	26332	22906	26307	22634	24949
Russian Federation	4518	1890	1608	1892	1503	1766	1225
Saudi Arabia	2074	1152	1142	1151	911	979	723
South Africa	1396	469	463	465	452	459	426
Thailand	1826	686	679	683	674	678	650
Tunisia, Morocco & West. Sahara	591	296	382	277	340	270	272
Turkey	730	431	487	431	457	407	395
Ukraine	798	275	334	275	329	265	292
United States	9513	4210	4647	4212	4570	4193	4410
Vietnam	1341	650	859	650	856	646	816

Supplementary Table 19: Black carbon emissions by region, climate scenario, and year under Fixed air pollution legislation. Source: POLES model.

Black carbon (kt)		Reference	Reference	INDC	INDC	2C	2C
Fixed air pollution legislation	2010	2030	2050	2030	2050	2030	2050
World	6584	7831	8548	7682	7656	7037	5764
Algeria and Libya	55	92	112	90	106	79	65
Argentina	46	48	53	48	50	46	40
Australia	34	35	38	35	35	34	33
Brazil	192	188	194	187	184	172	139
Canada	48	70	115	66	70	56	56
Chile	26	29	30	28	27	27	24
China	1451	1681	1531	1579	1218	1443	921
Iceland, Norway and Switzerland	10	7	6	7	6	7	5
Egypt	56	78	111	78	105	69	69
European Union (28)	329	262	249	250	212	248	168
India	1106	1381	1430	1382	1256	1128	739
Indonesia	289	316	319	317	311	302	254
Iran	75	90	104	89	95	76	61
Japan	35	41	50	42	45	42	41
Korea (Republic)	45	42	37	38	30	38	27
Malaysia	41	53	61	53	58	51	50
Mediterranean Middle-East	20	27	39	25	35	24	23
Mexico	94	89	96	86	88	84	68
New Zealand	5	6	6	5	5	5	5
Rest of Balkans	15	12	12	12	11	11	9
Rest of Central America & Carib.	74	83	95	82	92	80	80
Rest of CIS	73	82	92	81	88	78	82
Rest of Pacific	8	13	44	13	31	11	13
Rest of Persian Gulf	75	107	125	106	117	95	84
Rest of South America	98	125	159	125	148	116	113
Rest of South Asia	288	388	522	389	504	370	335
Rest of South-East Asia	156	165	168	165	163	156	140
Rest of Sub-Saharan Africa	969	1298	1609	1294	1580	1247	1300
Russian Federation	197	210	185	210	172	198	144
Saudi Arabia	73	101	116	100	94	82	66
South Africa	65	77	72	73	62	69	47
Thailand	83	93	98	89	88	85	69
Tunisia, Morocco & West. Sahara	36	55	75	50	60	49	46
Turkey	75	78	85	78	62	61	42
Ukraine	32	26	23	26	22	24	17
United States	241	305	397	304	343	302	319
Vietnam	69	80	88	80	85	76	66

Supplementary Table 20: Black carbon emissions by region, climate scenario, and year under Stringent air pollution legislation. Source: POLES model.

Black carbon (kt)		Reference	Reference	INDC	INDC	2C	2C
Stringent air pollution legislation	2010	2030	2050	2030	2050	2030	2050
World	6584	6531	4319	6410	3943	5933	3201
Algeria and Libya	55	91	37	90	36	79	26
Argentina	46	44	30	44	29	41	23
Australia	34	30	17	30	15	29	14
Brazil	192	147	82	146	77	133	57
Canada	48	57	68	53	33	43	22
Chile	26	25	14	25	13	24	11
China	1451	1314	671	1226	511	1110	386
Iceland, Norway and Switzerland	10	4	2	4	2	4	2
Egypt	56	77	39	77	37	69	27
European Union (28)	329	171	84	162	72	161	63
India	1106	1027	457	1028	426	881	328
Indonesia	289	289	172	289	170	282	150
Iran	75	76	45	75	42	64	26
Japan	35	27	23	27	22	28	20
Korea (Republic)	45	26	12	23	10	23	10
Malaysia	41	49	22	49	21	47	19
Mediterranean Middle-East	20	20	14	20	13	19	10
Mexico	94	73	41	69	37	68	29
New Zealand	5	5	3	4	2	4	2
Rest of Balkans	15	10	4	9	4	9	3
Rest of Central America & Carib.	74	79	55	79	54	76	50
Rest of CIS	73	78	51	77	49	75	45
Rest of Pacific	8	13	34	13	23	10	8
Rest of Persian Gulf	75	88	45	88	43	79	32
Rest of South America	98	105	68	105	64	98	48
Rest of South Asia	288	352	154	352	153	336	122
Rest of South-East Asia	156	149	89	149	88	143	83
Rest of Sub-Saharan Africa	969	1253	1517	1250	1492	1209	1245
Russian Federation	197	192	77	192	71	182	59
Saudi Arabia	73	86	48	85	38	70	27
South Africa	65	72	35	68	31	64	23
Thailand	83	86	43	83	38	79	30
Tunisia, Morocco & West. Sahara	36	55	27	50	21	49	17
Turkey	75	65	31	65	23	49	16
Ukraine	32	24	18	24	17	22	14
United States	241	200	145	206	130	205	124
Vietnam	69	72	39	73	38	70	30

Supplementary Table 21: Black carbon emissions by region, climate scenario, and year under Best available air pollution abatement technologies. Source: POLES model.

Black carbon (kt)		Reference	Reference	INDC	INDC	2C	2C
Best available abatement technologies	2010	2030	2050	2030	2050	2030	2050
World	6584	1409	1374	1370	1218	1302	1077
Algeria and Libya	55	5	6	5	6	5	6
Argentina	46	15	14	15	14	15	13
Australia	34	7	6	7	6	7	6
Brazil	192	29	26	29	26	28	24
Canada	48	25	53	21	20	13	10
Chile	26	6	6	6	5	6	5
China	1451	457	336	425	253	385	199
Iceland, Norway and Switzerland	10	2	1	2	1	2	1
Egypt	56	7	9	7	9	7	9
European Union (28)	329	56	45	54	41	53	36
India	1106	144	125	144	120	138	105
Indonesia	289	48	45	48	45	48	42
Iran	75	9	10	9	9	8	7
Japan	35	11	15	11	14	11	13
Korea (Republic)	45	6	5	6	5	6	5
Malaysia	41	5	5	5	5	5	5
Mediterranean Middle-East	20	3	4	3	4	3	4
Mexico	94	16	16	16	16	16	14
New Zealand	5	1	1	1	1	1	1
Rest of Balkans	15	2	2	2	2	2	1
Rest of Central America & Carib.	74	16	16	16	16	15	15
Rest of CIS	73	20	22	19	22	19	22
Rest of Pacific	8	5	31	5	20	4	6
Rest of Persian Gulf	75	9	11	9	11	9	9
Rest of South America	98	16	20	16	19	16	17
Rest of South Asia	288	45	42	45	42	44	38
Rest of South-East Asia	156	29	28	29	28	28	28
Rest of Sub-Saharan Africa	969	248	305	248	304	247	296
Russian Federation	197	38	32	38	31	36	28
Saudi Arabia	73	12	12	11	11	10	9
South Africa	65	11	9	10	8	10	7
Thailand	83	12	10	12	10	11	9
Tunisia, Morocco & West. Sahara	36	4	4	3	4	3	4
Turkey	75	13	11	13	9	11	8
Ukraine	32	6	5	6	5	6	5
United States	241	63	74	64	69	64	65
Vietnam	69	10	9	10	9	10	7

Supplementary Table 22: Organic carbon emissions by region, climate scenario, and year under Fixed air pollution legislation. Source: POLES model.

Organic carbon (kt)		Reference	Reference	INDC	INDC	2C	2C
Fixed air pollution legislation	2010	2030	2050	2030	2050	2030	2050
World	14014	15839	16600	15697	15665	15208	13932
Algeria and Libya	76	126	154	124	147	112	105
Argentina	128	130	137	130	135	131	131
Australia	51	59	71	59	67	59	67
Brazil	396	387	384	386	378	377	361
Canada	70	102	159	99	120	91	109
Chile	72	74	76	73	72	72	69
China	3526	3731	3169	3614	2751	3449	2217
Iceland, Norway and Switzerland	13	10	10	10	10	10	9
Egypt	105	143	193	143	189	135	159
European Union (28)	459	414	396	401	351	398	312
India	1978	2042	1866	2043	1743	1922	1395
Indonesia	652	727	716	727	714	722	662
Iran	90	105	120	105	113	92	86
Japan	36	70	94	70	88	72	84
Korea (Republic)	69	78	75	74	66	74	61
Malaysia	61	81	95	81	94	80	87
Mediterranean Middle-East	34	47	69	46	64	44	54
Mexico	192	193	206	190	200	189	179
New Zealand	9	10	11	9	10	9	10
Rest of Balkans	30	25	25	25	25	24	22
Rest of Central America & Carib.	219	235	257	235	255	232	240
Rest of CIS	159	193	251	192	244	190	255
Rest of Pacific	19	27	79	27	58	23	28
Rest of Persian Gulf	74	102	127	102	123	95	106
Rest of South America	246	297	371	297	358	291	329
Rest of South Asia	728	700	749	701	744	687	615
Rest of South-East Asia	341	385	399	386	397	380	383
Rest of Sub-Saharan Africa	2688	3606	4410	3603	4391	3579	4225
Russian Federation	302	311	284	311	267	294	234
Saudi Arabia	76	89	100	89	83	77	67
South Africa	139	150	131	147	122	144	102
Thailand	148	163	158	159	149	155	129
Tunisia, Morocco & West. Sahara	67	84	102	80	88	79	77
Turkey	129	135	151	135	117	109	90
Ukraine	82	67	57	67	56	64	51
United States	397	563	767	580	704	577	680
Vietnam	153	173	180	173	175	169	145

Supplementary Table 23: Organic carbon emissions by region, climate scenario, and year under Stringent air pollution legislation. Source: POLES model.

Organic carbon (kt)		Reference	Reference	INDC	INDC	2C	2C
Stringent air pollution legislation	2010	2030	2050	2030	2050	2030	2050
World	14014	14358	10048	14231	9656	13810	8923
Algeria and Libya	76	115	63	113	61	103	55
Argentina	128	125	87	125	85	126	82
Australia	51	49	30	49	29	49	28
Brazil	396	356	191	355	187	345	172
Canada	70	91	81	88	52	80	43
Chile	72	68	35	68	34	66	33
China	3526	3121	1436	3017	1259	2873	1048
Iceland, Norway and Switzerland	13	9	5	8	5	8	5
Egypt	105	137	109	137	108	130	101
European Union (28)	459	335	181	325	166	323	146
India	1978	1869	1037	1869	995	1768	878
Indonesia	652	689	358	690	356	685	338
Iran	90	85	53	85	50	75	39
Japan	36	53	39	53	38	55	36
Korea (Republic)	69	69	30	67	28	67	27
Malaysia	61	78	38	78	38	77	36
Mediterranean Middle-East	34	40	29	39	27	37	24
Mexico	192	170	102	168	99	167	91
New Zealand	9	8	5	8	5	8	5
Rest of Balkans	30	21	9	21	9	20	8
Rest of Central America & Carib.	219	216	146	216	145	213	140
Rest of CIS	159	181	130	179	127	178	129
Rest of Pacific	19	24	56	23	38	20	14
Rest of Persian Gulf	74	82	55	82	53	77	47
Rest of South America	246	273	156	273	152	268	138
Rest of South Asia	728	665	373	666	372	655	342
Rest of South-East Asia	341	372	221	373	221	368	218
Rest of Sub-Saharan Africa	2688	3557	4146	3555	4128	3532	3980
Russian Federation	302	294	139	295	132	279	118
Saudi Arabia	76	69	49	69	43	61	37
South Africa	139	140	61	137	58	135	49
Thailand	148	156	73	153	69	149	62
Tunisia, Morocco & West. Sahara	67	83	55	78	48	77	45
Turkey	129	123	63	124	50	98	41
Ukraine	82	64	44	64	43	61	41
United States	397	403	285	416	269	414	260
Vietnam	153	165	79	165	78	162	68

Supplementary Table 24: Organic carbon emissions by region, climate scenario, and year under Best available air pollution abatement technologies. Source: POLES model.

Organic carbon (kt)		Reference	Reference	INDC	INDC	2C	2C
Best available abatement technologies	2010	2030	2050	2030	2050	2030	2050
World	14014	4335	4668	4297	4488	4214	4237
Algeria and Libya	76	28	34	28	33	28	31
Argentina	128	72	65	72	65	72	65
Australia	51	18	17	18	16	18	16
Brazil	396	128	117	128	116	127	113
Canada	70	29	50	26	25	20	17
Chile	72	23	22	23	21	23	21
China	3526	1135	899	1102	802	1058	696
Iceland, Norway and Switzerland	13	4	3	3	3	3	3
Egypt	105	40	57	40	57	40	55
European Union (28)	459	121	110	118	104	118	93
India	1978	515	526	515	515	504	487
Indonesia	652	149	152	149	151	149	147
Iran	90	27	29	27	28	25	24
Japan	36	21	24	21	23	22	22
Korea (Republic)	69	20	17	20	16	20	16
Malaysia	61	20	21	20	21	20	21
Mediterranean Middle-East	34	12	17	12	16	12	15
Mexico	192	65	70	65	69	65	66
New Zealand	9	3	3	3	3	3	3
Rest of Balkans	30	5	5	5	4	5	4
Rest of Central America & Carib.	219	63	66	63	66	63	64
Rest of CIS	159	68	78	67	77	67	78
Rest of Pacific	19	9	49	9	32	6	10
Rest of Persian Gulf	74	27	34	27	33	26	30
Rest of South America	246	71	82	71	81	70	77
Rest of South Asia	728	178	201	178	201	177	193
Rest of South-East Asia	341	100	112	100	112	100	111
Rest of Sub-Saharan Africa	2688	932	1372	932	1370	930	1355
Russian Federation	302	90	77	90	76	89	72
Saudi Arabia	76	35	34	35	31	33	28
South Africa	139	37	32	37	31	36	28
Thailand	148	40	36	40	36	39	35
Tunisia, Morocco & West. Sahara	67	20	24	20	23	20	23
Turkey	129	34	32	34	28	30	26
Ukraine	82	22	19	22	19	22	18
United States	397	145	154	147	148	147	143
Vietnam	153	29	33	29	32	29	30

Supplementary Table 25: Carbon monoxide emissions by region, climate scenario, and year under Fixed air pollution legislation. Source: POLES model.

Carbon monoxide (kt)		Reference	Reference	INDC	INDC	2C	2C
Fixed air pollution legislation	2010	2030	2050	2030	2050	2030	2050
World	1001025	1004461	962991	996630	916521	968809	821283
Algeria and Libya	4159	7819	9788	7693	9355	6740	5727
Argentina	9106	8216	7436	8216	7398	8144	7030
Australia	8715	7635	6801	7632	6680	7564	6526
Brazil	24708	21877	20142	21855	19926	21385	18270
Canada	5302	11710	22036	10455	9309	7271	5299
Chile	2772	2931	2780	2899	2674	2836	2406
China	166846	199208	170122	193643	151568	187220	128308
Iceland, Norway and Switzerland	701	572	505	568	512	565	484
Egypt	4398	6149	8721	6162	8563	5670	6617
European Union (28)	23515	19892	19115	19419	17906	19293	15399
India	68459	71111	67764	71175	63171	65640	49606
Indonesia	46622	46418	45684	46458	45634	45508	41332
Iran	4954	5685	6172	5697	5924	4908	4048
Japan	4527	4681	4571	4754	4351	4703	4160
Korea (Republic)	3878	4441	4070	4227	3813	4234	3748
Malaysia	4902	6480	7507	6486	7412	6272	6603
Mediterranean Middle-East	2096	2381	2946	2318	2821	2217	2188
Mexico	10360	9288	9508	9150	9327	9048	8118
New Zealand	530	516	505	485	458	484	435
Rest of Balkans	897	716	671	713	657	678	578
Rest of Central America & Carib.	8894	9236	9906	9221	9802	8999	8811
Rest of CIS	8553	9152	9405	9046	9382	8804	8223
Rest of Pacific	638	821	1446	824	1339	800	1213
Rest of Persian Gulf	4780	6172	7017	6175	6764	5456	5015
Rest of South America	20604	20786	20512	20796	20336	20123	17932
Rest of South Asia	20763	24174	29763	24203	29361	23114	23161
Rest of South-East Asia	35101	32503	29718	32506	29603	32051	28327
Rest of Sub-Saharan Africa	402914	365580	338712	365470	338259	364351	329133
Russian Federation	17515	15343	13558	15375	12714	14311	10312
Saudi Arabia	4679	5741	6543	5741	4961	4631	3605
South Africa	8893	9062	8309	8870	7960	8672	6847
Thailand	11671	12134	12092	11969	11903	11778	10662
Tunisia, Morocco & West. Sahara	1912	2478	3096	2341	2734	2305	2319
Turkey	4350	4486	5130	4500	4546	3980	3968
Ukraine	4434	3191	2729	3195	2701	3100	2371
United States	38323	35543	36872	36057	35497	35858	32610
Vietnam	9550	10334	11342	10337	11199	10095	9891

Supplementary Table 26: Carbon monoxide emissions by region, climate scenario, and year under Stringent air pollution legislation. Source: POLES model.

Carbon monoxide (kt)		Reference	Reference	INDC	INDC	2C	2C
Stringent air pollution legislation	2010	2030	2050	2030	2050	2030	2050
World	1001025	895940	658991	889004	630601	867153	589814
Algeria and Libya	4159	5819	2857	5728	2754	5054	2007
Argentina	9106	7347	5572	7345	5547	7312	5402
Australia	8715	6973	5599	6968	5519	6917	5463
Brazil	24708	20094	15142	20075	14990	19737	14271
Canada	5302	11200	19995	9937	7350	6773	3449
Chile	2772	2302	1445	2280	1392	2239	1285
China	166846	156498	76884	151730	67672	146320	56559
Iceland, Norway and Switzerland	701	480	328	476	332	474	318
Egypt	4398	5084	3548	5093	3496	4729	3034
European Union (28)	23515	16795	11928	16444	11250	16340	9737
India	68459	61707	32236	61765	31311	57551	28201
Indonesia	46622	41003	22813	41030	22750	40416	21959
Iran	4954	3899	3030	3903	2887	3381	2158
Japan	4527	4172	2849	4231	2699	4180	2571
Korea (Republic)	3878	4109	2457	3915	2303	3923	2279
Malaysia	4902	4914	2688	4917	2655	4781	2481
Mediterranean Middle-East	2096	1654	1426	1619	1383	1562	1188
Mexico	10360	7452	5250	7356	5162	7290	4736
New Zealand	530	410	320	389	289	388	281
Rest of Balkans	897	589	343	586	335	560	310
Rest of Central America & Carib.	8894	8009	4833	7993	4809	7827	4566
Rest of CIS	8553	7943	5533	7848	5504	7645	4917
Rest of Pacific	638	761	1089	764	963	741	813
Rest of Persian Gulf	4780	4267	3542	4264	3426	3812	2791
Rest of South America	20604	17258	12687	17258	12551	16841	11654
Rest of South Asia	20763	21951	10407	21975	10228	21073	8840
Rest of South-East Asia	35101	30261	20869	30261	20847	29948	20540
Rest of Sub-Saharan Africa	402914	361186	327414	361093	326986	360183	319732
Russian Federation	17515	11847	7040	11866	6603	11149	5641
Saudi Arabia	4679	3807	3079	3802	2438	3122	1881
South Africa	8893	7062	4399	6913	4241	6769	3844
Thailand	11671	10315	6247	10204	6163	10073	5846
Tunisia, Morocco & West. Sahara	1912	2232	1407	2107	1305	2072	1197
Turkey	4350	4000	3130	4011	2830	3508	2531
Ukraine	4434	2729	1535	2732	1509	2656	1359
United States	38323	30801	23579	31114	22673	30952	20896
Vietnam	9550	9011	5493	9012	5448	8855	5076

Supplementary Table 27: Carbon monoxide emissions by region, climate scenario, and year under Best available air pollution abatement technologies. Source: POLES model.

Carbon monoxide (kt)		Reference	Reference	INDC	INDC	2C	2C
Best available abatement technologies	2010	2030	2050	2030	2050	2030	2050
World	1001025	534846	471795	531203	449728	522402	428777
Algeria and Libya	4159	988	1177	975	1136	899	900
Argentina	9106	5488	4663	5488	4657	5478	4611
Australia	8715	5988	5088	5982	5016	5940	4940
Brazil	24708	14307	12132	14297	12081	14169	11745
Canada	5302	8617	18607	7330	5984	4187	2111
Chile	2772	899	820	892	792	880	750
China	166846	57631	47208	55782	41727	53596	35420
Iceland, Norway and Switzerland	701	260	215	257	218	255	201
Egypt	4398	1212	1639	1212	1622	1172	1478
European Union (28)	23515	8929	8103	8787	7771	8721	6505
India	68459	24069	21336	24070	20732	23058	18953
Indonesia	46622	21748	18589	21749	18550	21653	18225
Iran	4954	1515	1661	1515	1580	1332	1279
Japan	4527	2330	1941	2368	1796	2267	1656
Korea (Republic)	3878	1415	1205	1317	1110	1314	1093
Malaysia	4902	1650	1594	1649	1582	1626	1526
Mediterranean Middle-East	2096	780	924	767	898	746	795
Mexico	10360	3704	3450	3670	3414	3646	3159
New Zealand	530	250	237	235	215	234	205
Rest of Balkans	897	278	240	277	236	267	216
Rest of Central America & Carib.	8894	2503	2400	2499	2381	2470	2278
Rest of CIS	8553	3208	3032	3187	3005	3152	2837
Rest of Pacific	638	381	864	381	718	355	527
Rest of Persian Gulf	4780	1863	2213	1857	2137	1684	1829
Rest of South America	20604	9975	8980	9966	8879	9841	8519
Rest of South Asia	20763	5213	5134	5212	5065	5093	4541
Rest of South-East Asia	35101	21171	17742	21155	17729	21071	17610
Rest of Sub-Saharan Africa	402914	290704	245876	290678	245754	290534	244913
Russian Federation	17515	5191	4379	5195	4149	4912	3724
Saudi Arabia	4679	1570	1680	1563	1409	1359	1160
South Africa	8893	3630	3063	3593	2979	3550	2781
Thailand	11671	5627	4800	5601	4774	5573	4662
Tunisia, Morocco & West. Sahara	1912	530	627	515	591	509	550
Turkey	4350	2238	2332	2242	2179	2109	1983
Ukraine	4434	1058	917	1058	898	1027	825
United States	38323	13643	13073	13596	12135	13488	10630
Vietnam	9550	4283	3857	4283	3829	4235	3643

Supplementary Table 28: Methane emissions by region, climate scenario, and year. Source: POLES model.

CH_4 (kt)	2010	Reference 2030	Reference 2050	INDC 2030	INDC 2050	2C 2030	2C 2050
World	341985	399160	452612	330136	285104	243793	158584
Algeria and Libya	3060	2817	3077	2011	1937	1762	1107
Argentina	4057	4522	4999	4520	3429	3055	2255
Australia	4869	3754	4225	3733	3119	3262	2027
Brazil	17023	19535	21059	18309	14962	11788	8358
Canada	4218	3299	3687	3042	2843	2512	1317
Chile	854	1137	1291	830	805	743	517
China	78306	96787	92587	55349	47249	48620	28312
Iceland, Norway and Switzerland	413	321	314	315	314	312	282
Egypt	2303	2795	3886	2793	2656	1972	1268
European Union (28)	19468	13823	12196	11390	9645	11384	6471
India	29287	38734	47056	38730	34778	26140	21476
Indonesia	10188	13202	14327	13190	10684	8898	6189
Iran	5490	6718	8184	6607	4967	4089	3074
Japan	983	932	860	899	613	743	481
Korea (Republic)	1523	1559	1589	1293	1182	1293	920
Malaysia	1559	1850	1552	1847	903	1116	493
Mediterranean Middle-East	899	1001	1823	657	1079	556	496
Mexico	7884	6076	5991	5349	5070	4931	2629
New Zealand	1357	1482	1591	991	994	991	724
Rest of Balkans	531	559	594	455	396	357	213
Rest of Central America & Carib.	3060	3815	4597	3812	3521	2678	1765
Rest of CIS	9192	8490	9643	7905	8378	7082	4081
Rest of Pacific	214	254	279	254	211	171	118
Rest of Persian Gulf	6216	9503	11685	9414	6640	5214	3729
Rest of South America	9987	11421	12832	11366	8700	7264	5075
Rest of South Asia	14600	19929	26783	19934	21075	14744	11417
Rest of South-East Asia	9333	15997	16268	15731	11432	9123	5772
Rest of Sub-Saharan Africa	21635	33435	56112	28392	36573	22525	14022
Russian Federation	23391	23292	26025	23222	9461	9846	4159
Saudi Arabia	2867	4168	4924	4060	2472	2154	1454
South Africa	3003	3757	4365	2350	2269	2050	1034
Thailand	4621	5510	6226	3935	3951	3379	2006
Tunisia, Morocco & West. Sahara	877	1401	2025	979	1213	966	772
Turkey	2720	3242	3516	3243	1979	1985	743
Ukraine	3164	2269	1878	2247	1161	1235	535
United States	27646	26012	27683	15221	13538	15135	10902
Vietnam	5186	5763	6881	5763	4906	3719	2389

Supplementary Table 29: Concentration of particulate matter with diameter smaller than 2.5 μgm^{-3} by region, climate scenario, and year under Fixed air pollution legislation.

$PM_{2.5}$ (μgm^{-3})		Reference	Reference	NDC	NDC	2C	2C
Fixed air pollution legislation	2010	2030	2050	2030	2050	2030	2050
World	42	51	58	50	51	46	39
Algeria and Libya	33	32	32	32	30	31	27
Argentina	14	13	13	13	13	13	12
Australia	6	6	5	6	5	6	5
Brazil	11	11	11	11	11	10	10
Canada	7	8	11	8	8	7	7
Chile	21	25	27	23	24	23	22
China	58	66	67	61	51	55	36
Iceland, Norway and Switzerland	10	9	9	9	8	8	7
Egypt	90	114	147	114	133	102	98
European Union (28)	15	13	13	12	12	12	9
India	65	95	118	94	100	81	64
Indonesia	14	14	14	14	14	14	13
Iran	48	54	58	54	56	49	47
Japan	12	13	13	12	11	12	10
Korea (Republic)	25	28	27	25	22	24	19
Malaysia	15	17	18	16	15	15	13
Mediterranean Middle-East	33	37	42	36	36	32	29
Mexico	20	21	23	21	22	20	21
New Zealand	5	5	5	5	5	5	5
Rest of Balkans	24	21	21	20	18	18	15
Rest of Central America & Carib.	22	23	25	23	24	22	22
Rest of CIS	28	32	36	33	37	31	32
Rest of Pacific	11	11	12	11	12	11	11
Rest of Persian Gulf	75	86	98	86	91	78	74
Rest of South America	21	22	26	22	24	21	21
Rest of South Asia	66	90	110	90	100	82	73
Rest of South-East Asia	30	32	32	33	32	30	26
Rest of Sub-Saharan Africa	40	40	41	40	39	40	38
Russian Federation	17	18	18	18	16	16	15
Saudi Arabia	130	137	143	136	136	130	124
South Africa	30	29	29	27	25	26	21
Thailand	22	23	23	23	21	21	18
Tunisia, Morocco & West. Sahara	24	28	30	27	27	25	22
Turkey	33	37	44	36	34	30	26
Ukraine	19	19	22	18	17	15	13
United States	9	10	11	9	10	9	9
Vietnam	27	29	30	28	27	26	22

Supplementary Table 30: Concentration of particulate matter with diameter smaller than 2.5 μgm^{-3} by region, climate scenario, and year under Stringent air pollution legislation.

$PM_{2.5}$ (μgm^{-3})		Reference	Reference	NDC	NDC	2C	2C
Stringent air pollution legislation	2010	2030	2050	2030	2050	2030	2050
World	42	48	39	47	36	43	30
Algeria and Libya	33	32	27	31	26	30	25
Argentina	14	13	11	13	11	13	11
Australia	6	6	5	6	5	6	5
Brazil	11	10	9	10	9	10	8
Canada	7	8	8	8	6	7	5
Chile	21	24	19	23	16	22	15
China	58	56	37	52	30	48	23
Iceland, Norway and Switzerland	10	8	7	8	6	7	6
Egypt	90	111	111	111	102	100	79
European Union (28)	15	11	9	11	8	10	7
India	65	89	69	89	62	76	46
Indonesia	14	14	12	14	12	14	12
Iran	48	52	46	52	46	48	41
Japan	12	12	9	11	9	11	8
Korea (Republic)	25	25	17	23	14	22	12
Malaysia	15	16	13	15	12	14	11
Mediterranean Middle-East	33	34	27	33	25	30	22
Mexico	20	20	16	19	16	19	15
New Zealand	5	5	5	5	5	5	5
Rest of Balkans	24	18	14	17	13	16	11
Rest of Central America & Carib.	22	22	21	22	21	22	20
Rest of CIS	28	31	27	32	28	30	25
Rest of Pacific	11	11	11	11	11	11	11
Rest of Persian Gulf	75	81	71	82	70	74	61
Rest of South America	21	21	20	21	19	20	18
Rest of South Asia	66	85	65	86	62	78	50
Rest of South-East Asia	30	30	25	31	25	29	22
Rest of Sub-Saharan Africa	40	40	39	39	38	40	37
Russian Federation	17	17	12	17	11	15	11
Saudi Arabia	130	132	123	132	121	127	116
South Africa	30	27	21	26	19	25	18
Thailand	22	22	17	22	16	20	14
Tunisia, Morocco & West. Sahara	24	26	22	25	21	24	18
Turkey	33	34	26	33	22	27	18
Ukraine	19	18	15	16	12	14	10
United States	9	9	8	8	7	8	7
Vietnam	27	27	21	27	20	25	17

Supplementary Table 31: Concentration of particulate matter with diameter smaller than 2.5 μgm^{-3} by region, climate scenario, and year under Best available air pollution abatement technologies.

$PM_{2.5}$ (μgm^{-3})<br>*Best available abatement technologies*	2010	Reference 2030	Reference 2050	NDC 2030	NDC 2050	2C 2030	2C 2050
World	42	26	27	25	26	24	24
Algeria and Libya	33	25	23	25	23	25	23
Argentina	14	11	10	11	10	11	10
Australia	6	5	5	5	5	5	5
Brazil	11	8	8	8	8	8	8
Canada	7	5	6	5	5	5	5
Chile	21	15	15	14	13	13	12
China	58	30	29	28	24	26	19
Iceland, Norway and Switzerland	10	6	6	6	6	6	5
Egypt	90	55	63	55	60	52	53
European Union (28)	15	7	7	7	7	7	6
India	65	33	36	33	33	31	29
Indonesia	14	11	11	11	11	11	11
Iran	48	39	40	39	40	37	38
Japan	12	8	8	8	8	8	7
Korea (Republic)	25	14	13	13	12	12	10
Malaysia	15	9	9	10	9	9	9
Mediterranean Middle-East	33	20	21	20	20	20	19
Mexico	20	13	14	13	13	13	13
New Zealand	5	5	5	5	5	5	5
Rest of Balkans	24	11	10	10	10	10	10
Rest of Central America & Carib.	22	18	19	18	18	18	18
Rest of CIS	28	22	23	22	23	21	21
Rest of Pacific	11	10	11	10	11	10	11
Rest of Persian Gulf	75	56	58	57	57	55	54
Rest of South America	21	17	18	17	18	17	17
Rest of South Asia	66	32	34	32	33	30	30
Rest of South-East Asia	30	21	20	21	20	20	19
Rest of Sub-Saharan Africa	40	35	34	35	33	35	34
Russian Federation	17	10	10	10	9	10	9
Saudi Arabia	130	113	114	113	113	112	112
South Africa	30	19	18	18	17	18	16
Thailand	22	14	13	14	12	13	12
Tunisia, Morocco & West. Sahara	24	16	16	16	16	16	15
Turkey	33	18	19	18	17	17	16
Ukraine	19	8	8	8	7	7	7
United States	9	6	6	6	6	6	6
Vietnam	27	17	16	16	15	16	14

Supplementary Table 32: Ozone mixing ratio by region, climate scenario, and year under Fixed air pollution legislation.

Ozone (M6M, ppb)		Reference	Reference	NDC	NDC	2C	2C
Fixed air pollution legislation	2010	2030	2050	2030	2050	2030	2050
World	59	68	75	66	69	62	60
Algeria and Libya	66	80	90	77	81	71	66
Argentina	37	39	40	38	37	36	33
Australia	35	36	37	35	35	33	32
Brazil	48	50	53	49	49	46	43
Canada	55	58	61	57	56	54	51
Chile	41	44	45	43	42	41	39
China	61	69	73	67	65	63	57
Iceland, Norway and Switzerland	53	55	57	53	52	51	47
Egypt	67	77	89	74	82	69	68
European Union (28)	56	58	61	56	56	54	50
India	69	90	107	89	100	82	81
Indonesia	40	44	48	43	44	41	39
Iran	70	80	86	78	78	70	64
Japan	58	63	66	61	60	58	55
Korea (Republic)	59	65	68	64	64	61	58
Malaysia	43	52	59	50	52	46	44
Mediterranean Middle-East	68	75	82	72	74	68	63
Mexico	60	62	66	60	60	57	54
New Zealand	33	34	35	33	33	31	30
Rest of Balkans	61	63	65	61	60	58	54
Rest of Central America & Carib.	46	50	54	49	50	46	44
Rest of CIS	57	61	65	60	61	56	52
Rest of Pacific	29	31	33	30	29	28	26
Rest of Persian Gulf	68	80	87	78	78	69	63
Rest of South America	42	45	48	44	45	42	39
Rest of South Asia	62	84	103	83	98	77	79
Rest of South-East Asia	49	54	56	52	52	49	46
Rest of Sub-Saharan Africa	55	59	66	57	61	56	56
Russian Federation	46	48	50	47	46	45	42
Saudi Arabia	63	75	84	73	75	65	59
South Africa	49	51	53	49	49	47	44
Thailand	49	56	61	54	56	52	49
Tunisia, Morocco & West. Sahara	59	69	75	66	68	61	56
Turkey	64	69	74	67	67	63	59
Ukraine	56	57	59	55	54	53	49
United States	67	71	74	69	68	66	63
Vietnam	46	53	56	51	50	48	44

Supplementary Table 33: Ozone mixing ratio by region, climate scenario, and year under Stringent air pollution legislation.

Ozone (M6M, ppb)<br>*Stringent air pollution legislation*	2010	Reference 2030	Reference 2050	NDC 2030	NDC 2050	2C 2030	2C 2050
World	59	63	63	62	58	58	52
Algeria and Libya	66	74	72	71	65	66	56
Argentina	37	37	36	36	33	34	30
Australia	35	35	35	34	33	33	30
Brazil	48	47	46	46	42	43	38
Canada	55	55	55	54	51	51	48
Chile	41	42	41	41	39	39	36
China	61	58	54	57	50	54	46
Iceland, Norway and Switzerland	53	52	52	50	48	48	44
Egypt	67	71	72	69	66	64	57
European Union (28)	56	55	56	54	51	51	47
India	69	86	84	85	78	79	67
Indonesia	40	43	43	41	39	39	35
Iran	70	75	70	73	64	66	55
Japan	58	58	56	56	53	54	49
Korea (Republic)	59	58	58	58	55	56	52
Malaysia	43	49	48	47	43	43	38
Mediterranean Middle-East	68	70	68	68	62	64	55
Mexico	60	59	57	57	52	54	47
New Zealand	33	33	33	32	31	31	28
Rest of Balkans	61	59	59	57	55	55	50
Rest of Central America & Carib.	46	47	47	46	43	43	39
Rest of CIS	57	58	57	57	53	54	47
Rest of Pacific	29	30	31	29	28	27	25
Rest of Persian Gulf	68	74	69	72	63	64	53
Rest of South America	42	42	41	41	38	39	34
Rest of South Asia	62	80	78	79	74	74	63
Rest of South-East Asia	49	49	48	48	44	46	40
Rest of Sub-Saharan Africa	55	57	61	55	57	54	53
Russian Federation	46	46	45	45	42	42	39
Saudi Arabia	63	70	65	68	59	60	49
South Africa	49	49	48	48	45	45	41
Thailand	49	51	50	50	46	47	42
Tunisia, Morocco & West. Sahara	59	64	62	61	56	57	49
Turkey	64	65	63	63	58	59	52
Ukraine	56	54	54	53	50	50	46
United States	67	67	67	65	62	62	57
Vietnam	46	45	43	44	40	42	36

Supplementary Table 34: Ozone mixing ratio by region, climate scenario, and year under Best available air pollution abatement technologies.

Ozone (M6M, ppb)		Reference	Reference	NDC	NDC	2C	2C
Best available abatement technologies	2010	2030	2050	2030	2050	2030	2050
World	59	50	52	49	48	47	45
Algeria and Libya	66	57	60	55	54	51	48
Argentina	37	32	33	31	30	30	28
Australia	35	34	34	33	32	31	30
Brazil	48	40	41	39	38	37	36
Canada	55	52	54	51	50	48	47
Chile	41	38	39	37	36	36	34
China	61	49	50	47	46	45	43
Iceland, Norway and Switzerland	53	49	50	48	46	46	43
Egypt	67	56	59	54	54	51	49
European Union (28)	56	53	54	51	50	48	46
India	69	56	59	55	55	53	50
Indonesia	40	36	38	35	35	34	32
Iran	70	57	60	55	54	52	49
Japan	58	52	54	51	50	49	47
Korea (Republic)	59	56	57	55	54	53	51
Malaysia	43	33	34	32	31	30	29
Mediterranean Middle-East	68	59	62	57	56	54	51
Mexico	60	49	51	48	47	45	43
New Zealand	33	31	32	30	30	29	27
Rest of Balkans	61	55	56	53	52	51	49
Rest of Central America & Carib.	46	41	43	39	39	37	36
Rest of CIS	57	50	52	48	48	46	44
Rest of Pacific	29	28	30	27	27	26	24
Rest of Persian Gulf	68	55	58	53	52	49	47
Rest of South America	42	34	35	33	33	31	31
Rest of South Asia	62	51	53	49	49	47	45
Rest of South-East Asia	49	42	42	41	39	39	37
Rest of Sub-Saharan Africa	55	50	51	49	47	48	46
Russian Federation	46	42	44	41	40	39	38
Saudi Arabia	63	49	52	47	46	44	42
South Africa	49	44	45	43	42	41	39
Thailand	49	39	39	38	37	36	35
Tunisia, Morocco & West. Sahara	59	52	54	50	49	47	44
Turkey	64	57	59	55	54	53	50
Ukraine	56	50	51	48	47	47	44
United States	67	62	64	60	59	58	55
Vietnam	46	39	40	38	37	36	34

Supplementary Table 35: Premature mortality due to particulate matter with diameter smaller than 2.5 μgm^{-3} by region, climate scenario, and year under Fixed air pollution legislation.

Premature mortality due to $PM_{2.5}$ (1000)		Reference	Reference	NDC	NDC	2C	2C
Fixed air pollution legislation	2010	2030	2050	2030	2050	2030	2050
World	4081	5712	7846	5632	7372	5431	6598
Algeria and Libya	13	19	30	19	29	19	28
Argentina	18	20	25	20	24	20	24
Australia	4	5	6	5	6	5	6
Brazil	50	70	99	70	97	69	94
Canada	8	13	20	12	16	11	15
Chile	6	10	16	9	15	9	14
China	1058	1611	2130	1559	1914	1502	1663
Iceland, Norway and Switzerland	4	5	7	5	6	5	6
Egypt	54	79	124	79	120	76	110
European Union (28)	272	294	343	283	317	276	281
India	1008	1488	2120	1487	2011	1409	1720
Indonesia	67	98	138	97	135	96	132
Iran	33	53	98	53	96	52	91
Japan	59	77	74	76	67	72	63
Korea (Republic)	16	28	43	27	38	26	35
Malaysia	8	15	23	14	21	14	20
Mediterranean Middle-East	13	19	32	19	30	18	28
Mexico	28	43	68	42	67	42	65
New Zealand	1	1	1	1	1	1	1
Rest of Balkans	17	18	18	17	17	17	15
Rest of Central America & Carib.	29	39	55	39	54	38	52
Rest of CIS	61	81	108	81	108	79	101
Rest of Pacific	3	4	7	4	7	4	6
Rest of Persian Gulf	31	51	94	51	92	50	87
Rest of South America	38	55	84	55	81	54	76
Rest of South Asia	288	414	670	414	650	402	583
Rest of South-East Asia	103	146	199	147	195	142	179
Rest of Sub-Saharan Africa	338	391	520	390	510	389	500
Russian Federation	148	160	154	158	144	152	141
Saudi Arabia	7	15	30	15	29	15	29
South Africa	24	26	27	26	25	25	23
Thailand	32	47	62	47	58	45	52
Tunisia, Morocco & West. Sahara	13	19	29	19	28	18	25
Turkey	24	35	55	34	49	32	43
Ukraine	67	65	63	63	57	59	50
United States	98	137	183	132	166	130	159
Vietnam	41	62	95	61	90	59	81

Supplementary Table 36: Premature mortality due to particulate matter with diameter smaller than 2.5μgm^{-3} by region, climate scenario, and year under Stringent air pollution legislation.

Premature mortality due to $PM_{2.5}$ (1000)		Reference	Reference	NDC	NDC	2C	2C
Stringent air pollution legislation	2010	2030	2050	2030	2050	2030	2050
World	4081	5511	6552	5437	6224	5250	5701
Algeria and Libya	13	19	28	19	27	19	27
Argentina	18	20	23	20	22	20	22
Australia	4	5	5	5	5	5	5
Brazil	50	69	85	69	84	68	82
Canada	8	12	16	12	11	10	9
Chile	6	10	13	9	12	9	12
China	1058	1515	1678	1467	1521	1415	1339
Iceland, Norway and Switzerland	4	5	5	4	5	4	4
Egypt	54	78	114	78	112	76	103
European Union (28)	272	273	267	262	248	256	217
India	1008	1458	1763	1457	1691	1381	1501
Indonesia	67	96	127	96	126	96	125
Iran	33	52	90	53	90	51	87
Japan	59	72	57	70	54	67	53
Korea (Republic)	16	26	33	25	30	25	28
Malaysia	8	14	19	14	18	13	17
Mediterranean Middle-East	13	19	27	19	26	18	25
Mexico	28	41	58	41	57	41	56
New Zealand	1	1	1	1	1	1	1
Rest of Balkans	17	17	15	16	14	16	13
Rest of Central America & Carib.	29	38	51	38	50	38	50
Rest of CIS	61	79	97	80	96	77	92
Rest of Pacific	3	4	6	4	6	4	6
Rest of Persian Gulf	31	51	86	51	85	49	82
Rest of South America	38	54	73	54	72	53	70
Rest of South Asia	288	407	562	407	550	395	509
Rest of South-East Asia	103	143	175	144	173	139	163
Rest of Sub-Saharan Africa	338	389	506	388	500	387	494
Russian Federation	148	155	129	154	123	149	123
Saudi Arabia	7	15	29	15	28	15	28
South Africa	24	25	23	25	22	24	21
Thailand	32	46	51	45	49	44	45
Tunisia, Morocco & West. Sahara	13	19	25	19	25	18	23
Turkey	24	33	44	33	40	30	37
Ukraine	67	63	54	61	50	57	44
United States	98	127	136	122	122	121	116
Vietnam	41	60	80	60	77	58	72

Supplementary Table 37: Premature mortality due to particulate matter with diameter smaller than 2.5μgm^{-3} by region, climate scenario, and year under Best available air pollution abatement technologies.

Premature mortality due to $PM_{2.5}$ (1000) *Best available abatement technologies*	2010	Reference 2030	Reference 2050	NDC 2030	NDC 2050	2C 2030	2C 2050
World	4081	4151	5582	4096	5333	4011	5019
Algeria and Libya	13	17	26	17	26	17	26
Argentina	18	18	22	18	21	18	21
Australia	4	4	5	4	5	4	5
Brazil	50	59	78	59	77	58	76
Canada	8	6	13	6	8	5	6
Chile	6	7	12	7	11	7	10
China	1058	1154	1510	1122	1373	1085	1236
Iceland, Norway and Switzerland	4	3	4	3	4	3	3
Egypt	54	62	96	62	94	61	90
European Union (28)	272	199	227	188	208	184	175
India	1008	980	1353	980	1316	957	1235
Indonesia	67	86	119	86	119	86	119
Iran	33	48	86	48	86	47	84
Japan	59	53	51	53	48	51	46
Korea (Republic)	16	19	29	18	26	18	24
Malaysia	8	10	15	10	15	10	15
Mediterranean Middle-East	13	15	24	15	24	15	23
Mexico	28	34	53	34	52	34	51
New Zealand	1	1	1	1	1	1	1
Rest of Balkans	17	12	13	12	12	12	12
Rest of Central America & Carib.	29	35	48	35	48	35	48
Rest of CIS	61	67	89	67	89	66	86
Rest of Pacific	3	4	6	4	6	4	6
Rest of Persian Gulf	31	45	81	45	80	45	79
Rest of South America	38	48	70	48	69	48	68
Rest of South Asia	288	280	439	280	433	276	413
Rest of South-East Asia	103	118	158	118	156	115	149
Rest of Sub-Saharan Africa	338	364	472	363	469	365	470
Russian Federation	148	121	114	121	112	119	111
Saudi Arabia	7	14	28	14	28	14	28
South Africa	24	21	21	21	20	21	20
Thailand	32	35	43	34	42	34	40
Tunisia, Morocco & West. Sahara	13	15	22	15	21	15	21
Turkey	24	25	38	25	36	24	34
Ukraine	67	42	39	41	36	40	34
United States	98	81	109	75	94	72	87
Vietnam	41	47	69	47	67	46	65

Supplementary Table 38: Premature mortality due to tropospheric ozone by region, climate scenario, and year under Fixed air pollution legislation.

Premature mortality due to O_3 (1000)		Reference	Reference	NDC	NDC	2C	2C
Fixed air pollution legislation	2010	2030	2050	2030	2050	2030	2050
World	198	386	631	367	539	321	382
Algeria and Libya	0	1	1	0	1	0	1
Argentina	0	0	0	0	0	0	0
Australia	0	0	0	0	0	0	0
Brazil	2	3	5	3	4	2	2
Canada	1	1	1	1	1	1	1
Chile	0	0	0	0	0	0	0
China	53	106	154	97	120	85	85
Iceland, Norway and Switzerland	0	0	0	0	0	0	0
Egypt	1	2	3	2	3	1	2
European Union (28)	11	15	20	13	16	11	11
India	86	179	308	175	277	154	197
Indonesia	0	1	3	1	2	1	0
Iran	1	2	4	2	3	1	2
Japan	2	3	3	3	2	2	2
Korea (Republic)	0	1	2	1	2	1	1
Malaysia	0	0	1	0	0	0	0
Mediterranean Middle-East	0	1	1	0	1	0	1
Mexico	2	3	5	3	4	2	3
New Zealand	0	0	0	0	0	0	0
Rest of Balkans	0	0	0	0	0	0	0
Rest of Central America & Carib.	0	1	1	1	1	0	0
Rest of CIS	1	2	2	1	2	1	1
Rest of Pacific	0	0	0	0	0	0	0
Rest of Persian Gulf	0	1	2	1	2	1	1
Rest of South America	0	1	2	1	1	1	0
Rest of South Asia	12	27	57	27	53	23	38
Rest of South-East Asia	3	6	9	6	7	5	5
Rest of Sub-Saharan Africa	4	5	9	5	8	5	6
Russian Federation	1	1	1	1	1	1	0
Saudi Arabia	0	0	0	0	0	0	0
South Africa	0	1	1	1	1	0	0
Thailand	1	2	3	1	2	1	1
Tunisia, Morocco & West. Sahara	0	1	1	0	1	0	0
Turkey	1	2	4	2	3	2	2
Ukraine	1	1	1	1	1	1	0
United States	12	17	23	16	19	15	16
Vietnam	1	2	3	1	2	1	1

Supplementary Table 39: Premature mortality due to tropospheric ozone by region, climate scenario, and year under Stringent air pollution legislation.

Premature mortality due to O_3 (1000)		Reference	Reference	NDC	NDC	2C	2C
Stringent air pollution legislation	2010	2030	2050	2030	2050	2030	2050
World	198	324	400	308	334	269	237
Algeria and Libya	0	0	1	0	1	0	0
Argentina	0	0	0	0	0	0	0
Australia	0	0	0	0	0	0	0
Brazil	2	2	3	2	2	1	0
Canada	1	1	1	1	1	1	1
Chile	0	0	0	0	0	0	0
China	53	70	74	64	54	56	36
Iceland, Norway and Switzerland	0	0	0	0	0	0	0
Egypt	1	2	2	1	2	1	1
European Union (28)	11	13	16	11	12	10	8
India	86	167	211	162	186	144	136
Indonesia	0	1	1	1	0	0	0
Iran	1	2	3	2	2	1	1
Japan	2	2	2	2	2	2	1
Korea (Republic)	0	1	1	1	1	1	1
Malaysia	0	0	0	0	0	0	0
Mediterranean Middle-East	0	0	1	0	1	0	0
Mexico	2	2	3	2	3	2	2
New Zealand	0	0	0	0	0	0	0
Rest of Balkans	0	0	0	0	0	0	0
Rest of Central America & Carib.	0	0	1	0	0	0	0
Rest of CIS	1	1	2	1	1	1	1
Rest of Pacific	0	0	0	0	0	0	0
Rest of Persian Gulf	0	1	1	1	1	1	1
Rest of South America	0	1	1	0	0	0	0
Rest of South Asia	12	25	37	25	34	22	24
Rest of South-East Asia	3	5	5	4	4	4	3
Rest of Sub-Saharan Africa	4	5	8	5	6	4	5
Russian Federation	1	1	1	1	0	0	0
Saudi Arabia	0	0	0	0	0	0	0
South Africa	0	1	1	0	0	0	0
Thailand	1	1	1	1	1	1	0
Tunisia, Morocco & West. Sahara	0	0	1	0	0	0	0
Turkey	1	2	3	2	2	2	2
Ukraine	1	1	1	1	0	1	0
United States	12	15	18	14	15	13	13
Vietnam	1	1	1	1	0	0	0

Supplementary Table 40: Premature mortality due to tropospheric ozone by region, climate scenario, and year under Best available air pollution abatement technologies.

Premature mortality due to O_3 (1000)		Reference	Reference	NDC	NDC	2C	2C
Best available abatement technologies	2010	2030	2050	2030	2050	2030	2050
World	198	156	232	141	176	118	126
Algeria and Libya	0	0	0	0	0	0	0
Argentina	0	0	0	0	0	0	0
Australia	0	0	0	0	0	0	0
Brazil	2	1	1	0	0	0	0
Canada	1	1	1	1	1	1	1
Chile	0	0	0	0	0	0	0
China	53	38	55	33	37	27	23
Iceland, Norway and Switzerland	0	0	0	0	0	0	0
Egypt	1	1	1	1	1	1	1
European Union (28)	11	11	14	10	11	8	7
India	86	67	102	63	83	54	61
Indonesia	0	0	0	0	0	0	0
Iran	1	1	2	1	1	1	1
Japan	2	2	2	2	1	1	1
Korea (Republic)	0	1	1	1	1	1	1
Malaysia	0	0	0	0	0	0	0
Mediterranean Middle-East	0	0	1	0	0	0	0
Mexico	2	1	2	1	2	1	1
New Zealand	0	0	0	0	0	0	0
Rest of Balkans	0	0	0	0	0	0	0
Rest of Central America & Carib.	0	0	0	0	0	0	0
Rest of CIS	1	1	1	1	1	1	1
Rest of Pacific	0	0	0	0	0	0	0
Rest of Persian Gulf	0	0	1	0	1	0	0
Rest of South America	0	0	0	0	0	0	0
Rest of South Asia	12	8	15	8	12	6	9
Rest of South-East Asia	3	3	4	2	3	2	2
Rest of Sub-Saharan Africa	4	4	5	3	4	3	3
Russian Federation	1	0	1	0	0	0	0
Saudi Arabia	0	0	0	0	0	0	0
South Africa	0	0	0	0	0	0	0
Thailand	1	0	0	0	0	0	0
Tunisia, Morocco & West. Sahara	0	0	0	0	0	0	0
Turkey	1	1	2	1	2	1	1
Ukraine	1	1	1	0	0	0	0
United States	12	13	17	12	14	11	11
Vietnam	1	0	0	0	0	0	0

Supplementary Table 41: Economic valuation of air pollution-related changes (from Reference) in lost work days due to sickness by region, scenario and year.

Avoided lost work days co-benefit* (% of GDP)<br>*Fixed air pollution legislation*	NDC<br>2030	2°C<br>2030	NDC<br>2050	2°C<br>2050
World	0.00	0.01	0.02	0.04
Australia and New Zealand	0.00	0.00	0.01	0.01
Brazil	0.00	0.00	0.01	0.01
Canada	0.00	0.01	0.02	0.03
China	0.01	0.02	0.04	0.08
Europe	0.00	0.01	0.01	0.03
India	0.00	0.01	0.02	0.06
Japan	0.00	0.01	0.02	0.02
North Africa and Middle East	0.00	0.01	0.01	0.02
Rest of the World	0.00	0.01	0.01	0.02
Russian Federation	0.00	0.01	0.02	0.02
Ukraine, Belarus and Moldova	0.01	0.02	0.03	0.06
United States	0.00	0.01	0.01	0.02
Stringent air pollution legislation	NDC<br>2030	2°C<br>2030	NDC<br>2050	2°C<br>2050
World	0.00	0.01	0.01	0.03
Australia and New Zealand	0.00	0.00	0.00	0.01
Brazil	0.00	0.00	0.01	0.01
Canada	0.00	0.01	0.02	0.03
China	0.01	0.02	0.03	0.06
Europe	0.00	0.01	0.01	0.02
India	0.00	0.01	0.01	0.04
Japan	0.00	0.01	0.01	0.01
North Africa and Middle East	0.00	0.01	0.01	0.01
Rest of the World	0.00	0.01	0.01	0.01
Russian Federation	0.00	0.01	0.01	0.01
Ukraine, Belarus and Moldova	0.01	0.02	0.02	0.04
United States	0.00	0.01	0.01	0.02
Best available abatement technologies	NDC<br>2030	2°C<br>2030	NDC<br>2050	2°C<br>2050
World	0.00	0.01	0.01	0.02
Australia and New Zealand	0.00	0.00	0.00	0.01
Brazil	0.00	0.00	0.00	0.01
Canada	0.00	0.01	0.02	0.03
China	0.01	0.01	0.02	0.05
Europe	0.00	0.01	0.01	0.02
India	0.00	0.01	0.01	0.02
Japan	0.00	0.00	0.01	0.01
North Africa and Middle East	0.00	0.00	0.01	0.01
Rest of the World	0.00	0.00	0.01	0.01
Russian Federation	0.00	0.01	0.01	0.01
Ukraine, Belarus and Moldova	0.00	0.01	0.01	0.02
United States	0.00	0.01	0.01	0.02

* Value of co-benefits expressed in consumption-based welfare metric: Equivalent variation as percent of GDP

Supplementary Table 42: Global crop yield improvements due to climate policy-induced ozone reduction, expressed in percentage difference from the Reference in 2030 and 2050. Total aggregated agricultural productivity includes unaffected crops and animal products.

2030	***NDC***			***2°C***		
	FLE	***SLE***	***BAT***	***FLE***	***SLE***	***BAT***
Maize	0.70	0.50	0.36	1.53	1.11	0.78
Rice	0.28	0.16	0.09	0.83	0.55	0.21
Soy	1.11	0.99	0.82	2.73	2.42	1.83
Wheat	0.63	0.48	0.38	1.68	1.30	0.87
High sensitivity crops	1.52	1.20	1.02	4.31	3.46	2.42
Low sensitivity crops	0.29	0.21	0.16	0.68	0.51	0.37
Medium sensitivity crops	0.78	0.56	0.41	2.02	1.49	0.93
**Total agricultural productivity**						
(all products, including non-sensitive)	0.29	0.23	0.19	0.78	0.62	0.43

2050	***NDC***			***2°C***		
	FLE	***SLE***	***BAT***	***FLE***	***SLE***	***BAT***
Maize	1.98	1.14	0.93	3.63	1.99	1.54
Rice	1.09	0.47	0.25	2.50	1.02	0.42
Soy	3.08	2.39	2.07	6.63	4.66	3.61
Wheat	2.01	1.24	1.01	4.31	2.44	1.76
High sensitivity crops	4.97	3.30	2.72	11.82	7.08	4.94
Low sensitivity crops	0.83	0.51	0.43	1.59	0.91	0.73
Medium sensitivity crops	2.52	1.40	1.08	5.18	2.68	1.85
**Total agricultural productivity**						
(all products, including non-sensitive)	0.92	0.60	0.49	2.03	1.21	0.86

Supplementary Table 43: Value of statistical life across regions and over time

Value of statistical life<br>*Million US$2005*	Low<br>2005	Medium<br>2005	High<br>2005	Low<br>2030	Medium<br>2030	High<br>2030	Low<br>2050	Medium<br>2050	High<br>2050
World	0.7	1.5	2.2	1.2	2.4	3.5	1.7	3.3	4.9
Australia and New Zealand	1.8	3.6	5.4	2.4	4.7	7.0	2.9	5.9	8.7
Brazil	0.7	1.4	2.0	1.0	1.9	2.9	1.4	2.8	4.2
Canada	2.0	4.0	5.9	2.5	5.0	7.4	3.1	6.3	9.3
China	0.4	0.9	1.3	1.5	3.0	4.5	2.5	5.0	7.4
Europe	1.5	3.1	4.6	1.9	3.9	5.7	2.4	4.8	7.1
India	0.3	0.5	0.8	0.8	1.5	2.3	1.4	2.9	4.2
Japan	2.0	4.0	5.9	2.6	5.1	7.6	3.4	6.8	10.1
North Africa and Middle East	0.9	1.7	2.6	1.3	2.6	3.9	1.8	3.6	5.3
Rest of the World	0.5	0.9	1.4	0.7	1.5	2.1	1.0	2.1	3.1
Russian Federation	0.8	1.5	2.3	1.3	2.7	3.9	1.8	3.6	5.3
Ukraine, Belarus and Moldova	0.6	1.2	1.7	1.0	1.9	2.8	1.6	3.1	4.6
United States	**2.5**	**5.0**	**7.4**	3.1	6.2	9.2	3.8	7.5	11.1

Supplementary Table 44: Economic valuation of air pollution-related changes (from Reference) in crop yields by region, scenario and year.

Agricultural crop yield co-benefit* (% of GDP)	NDC	2°C	NDC	2°C
Fixed air pollution legislation	2030	2030	2050	2050
World	0.02	0.06	0.08	0.18
Australia and New Zealand	0.00	0.01	0.00	0.01
Brazil	0.02	0.05	0.04	0.10
Canada	0.01	0.02	0.02	0.04
China	0.07	0.16	0.20	0.37
Europe	0.01	0.03	0.04	0.08
India	0.06	0.25	0.25	0.76
Japan	0.01	0.02	0.02	0.05
North Africa and Middle East	0.04	0.10	0.11	0.23
Rest of the World	0.02	0.05	0.05	0.12
Russian Federation	0.02	0.05	0.06	0.11
Ukraine, Belarus and Moldova	0.02	0.04	0.05	0.12
United States	0.01	0.02	0.02	0.04
	NDC	2°C	NDC	2°C
Stringent air pollution legislation	2030	2030	2050	2050
World	0.02	0.05	0.05	0.10
Australia and New Zealand	0.00	0.01	0.00	0.01
Brazil	0.02	0.04	0.03	0.07
Canada	0.01	0.02	0.02	0.03
China	0.04	0.09	0.09	0.16
Europe	0.01	0.03	0.03	0.06
India	0.05	0.23	0.18	0.46
Japan	0.01	0.02	0.01	0.03
North Africa and Middle East	0.03	0.09	0.08	0.15
Rest of the World	0.01	0.04	0.04	0.07
Russian Federation	0.02	0.04	0.04	0.07
Ukraine, Belarus and Moldova	0.01	0.03	0.04	0.08
United States	0.01	0.02	0.02	0.03
	NDC	2°C	NDC	2°C
Best available abatement technologies	2030	2030	2050	2050
World	0.01	0.03	0.04	0.07
Australia and New Zealand	0.00	0.01	0.00	0.01
Brazil	0.01	0.03	0.03	0.05
Canada	0.01	0.01	0.01	0.03
China	0.03	0.07	0.07	0.13
Europe	0.01	0.02	0.03	0.04
India	0.04	0.11	0.12	0.22
Japan	0.01	0.01	0.01	0.02
North Africa and Middle East	0.03	0.06	0.07	0.11
Rest of the World	0.01	0.03	0.03	0.04
Russian Federation	0.01	0.03	0.04	0.05
Ukraine, Belarus and Moldova	0.01	0.02	0.03	0.07
United States	0.01	0.01	0.01	0.03

* Value of co-benefits expressed in consumption-based welfare metric: Equivalent variation as percent of GDP

Supplementary Table 45: Mapping of IPCC sectors to POLES-JRC sectors

IPCC sector	IPCC definition	POLES-JRC sector	POLES-JRC sub-sector
Energy	Energy production and distribution: Public electricity and heat production, petroleum refining, manufacture of solid fuels and other energy industries. Fugitive emissions from solid fuels, oil and gas.	Transformation	
Domestic	Residential and commercial combustion	Buildings	
Industry	Industrial processes and combustion: Manufacturing industries and construction, inc. production of metals, chemicals, pulp /paper / print, food processing/beverages/tobacco, and other industries.	Industry	
Agriculture	Agriculture (animals, rice, soil): Enteric fermentation, manure management, rice cultivation, soil emissions, other	Agriculture	
Transport	Land transport: Road transportation (cars, light duty trucks, heavy duty trucks and buses, motorcycles, evaporative emissions from vehicles), railways, and other transportation (pipeline transport, off- road).	Transport	Domestic Road<br><br>Other land-based transport
Shipping		Transport	International Maritime
Aviation		Transport	Aviation Domestic + International
Forest burning	Forest fires	Fires	Forest Fires
Agricultural Waste Burning	Agriculture (waste burning on fields): Field burning of agricultural residues	Fires	Agricultural Waste Burning
Savannah fires	Savanna burning, grassland fires	Fires	Savannah Burning<br><br>Peat Fires
Solvents	Solvent production and use	Solvents	
Waste	Waste Treatment and Disposal: Solid waste disposal on land, wastewater handling, waste incineration	Waste	

Supplementary Table 46: Population and baseline mortality rates by region for the years 2010, 2030, and 2050.

	Population (10^6)			Baseline mortality rate (10^{-3})		
	2010	2030	2050	2010	2030	2050
World	6897	8475	9717	7	7	8
Algeria and Libya	42	56	65	4	5	6
Argentina	41	49	55	7	7	8
Australia	22	28	33	6	7	8
Brazil	199	229	238	5	7	9
Canada	34	40	44	7	9	10
Chile	17	20	22	6	8	11
China	1342	1416	1349	6	8	11
Iceland, Norway and Switzerland	13	16	18	8	8	10
Egypt	82	117	151	6	6	6
European Union (28)	503	519	526	10	11	12
India	1231	1528	1705	7	8	9
Indonesia	242	295	322	6	7	9
Iran	74	89	92	4	6	10
Japan	127	120	107	10	13	14
Korea (Republic)	49	53	51	5	8	13
Malaysia	28	36	41	4	5	7
Mediterranean Middle-East	43	60	75	4	4	5
Mexico	119	148	164	5	6	8
New Zealand	4	5	6	7	8	10
Rest of Balkans	23	21	19	9	10	12
Rest of Central America & Carib.	83	100	112	6	6	8
Rest of CIS	88	105	114	7	8	9
Rest of Pacific	9	13	16	8	8	9
Rest of Persian Gulf	72	116	160	4	4	5
Rest of South America	140	172	191	4	5	6
Rest of South Asia	398	531	626	7	6	8
Rest of South-East Asia	199	250	285	7	7	9
Rest of Sub-Saharan Africa	824	1392	2136	8	6	5
Russian Federation	143	139	129	12	13	14
Saudi Arabia	28	39	46	2	3	5
South Africa	52	60	66	10	9	8
Thailand	67	68	62	7	10	14
Tunisia, Morocco and West. Sahara	43	52	57	5	6	7
Turkey	72	88	96	4	4	6
Ukraine	46	41	35	14	16	17
United States	310	356	390	8	9	10
Vietnam	88	105	113	6	7	10

Supplementary Table 47: Classification of FAO products with respect to ozone sensitivity

Sensitivity of yield to ground-level ozone mixing ratio		
**High**	**Medium**	**Low**
Sugar cane	Potatoes	Barley
Beans, dry	Sweet potatoes	Plums and sloes
Broad beans, horse beans, dry	Sugar beet	
Pulses, nes	Rapeseed	
Cottonseed	Cauliflowers and broccoli	
Lettuce and chicory	Grapes	
Tomatoes	Tobacco, unmanufactured	
Onions, shallots, green		
Onions, dry		
Beans, green		
String beans		
Carrots and turnips		
Watermelons		
Cotton lint		

Supplementary Table 48: Regional aggregation in JRC-GEM-E3

Code	Aggregate	Countries included:
AUZ	Australia and New Zealand	Australia, New Zealand
BRA	Brazil	Brazil
CAN	Canada	Canada
CHN	China	China
Europe	Europe	28 EU Member States, Switzerland, Norway, Iceland, Liechtenstein, Bosnia and Herzegovina, Republic of Macedonia, Montenegro, Serbia, Montenegro, Serbia, Turkey
IND	India	India
JPN	Japan	Japan
NAM	North Africa and Middle East	Bahrain, Iran, Israel, Jordan, Kuwait, Oman, Qatar, Saudi Arabia, United Arab Emirates, Iraq, Lebanon, Syria, Yemen, Egypt, Morocco, Tunisia, Algeria, Libya, Western Sahara
RUS	Russian Federation	Russian Federation
UBM	Ukraine, Belarus and Moldova	Ukraine, Belarus and Moldova
USA	United States	United States
ROW	Rest of the World	Remaining countries not included elsewhere
